# Synaptic Loss in Frontotemporal Dementia Revealed by [ ^11^C]UCB‐J Positron Emission Tomography

**DOI:** 10.1002/ana.26543

**Published:** 2022-11-16

**Authors:** Maura Malpetti, P. Simon Jones, Thomas E. Cope, Negin Holland, Michelle Naessens, Matthew A. Rouse, Timothy Rittman, George Savulich, David J. Whiteside, Duncan Street, Tim D. Fryer, Young T. Hong, Selena Milicevic Sephton, Franklin I. Aigbirhio, John T. O′Brien, James B. Rowe

**Affiliations:** ^1^ Department of Clinical Neurosciences University of Cambridge Cambridge UK; ^2^ Cambridge University Hospitals National Health Service Foundation Trust Cambridge UK; ^3^ Medical Research Council Cognition and Brain Sciences Unit University of Cambridge UK; ^4^ Department of Psychiatry University of Cambridge Cambridge UK; ^5^ Wolfson Brain Imaging Centre University of Cambridge Cambridge UK

## Abstract

**Objective:**

Synaptic loss is an early feature of neurodegenerative disease models, and is severe in post mortem clinical studies, including frontotemporal dementia. Positron emission tomography (PET) with radiotracers that bind to synaptic vesicle glycoprotein 2A enables quantification of synaptic density in vivo. This study used [^11^C]UCB‐J PET in participants with behavioral variant frontotemporal dementia (bvFTD), testing the hypothesis that synaptic loss is severe and related to clinical severity.

**Methods:**

Eleven participants with clinically probable bvFTD and 25 age‐ and sex‐matched healthy controls were included. Participants underwent dynamic [^11^C]UCB‐J PET, structural magnetic resonance imaging, and a neuropsychological battery, including the revised Addenbrooke Cognitive Examination, and INECO frontal screening. General linear models compared [^11^C]UCB‐J binding potential maps and gray matter volume between groups, and assessed associations between synaptic density and clinical severity in patients. Analyses were also performed using partial volume corrected [^11^C]UCB‐J binding potential from regions of interest (ROIs).

**Results:**

Patients with bvFTD showed severe synaptic loss compared to controls. [^11^C]UCB‐J binding was reduced bilaterally in medial and dorsolateral frontal regions, inferior frontal gyri, anterior and posterior cingulate gyrus, insular cortex, and medial temporal lobe. Synaptic loss in the frontal and cingulate regions correlated significantly with cognitive impairments. Synaptic loss was more severe than atrophy. Results from ROI‐based analyses mirrored the voxelwise results.

**Interpretation:**

In accordance with preclinical models, and human postmortem evidence, there is widespread frontotemporal loss of synapses in symptomatic bvFTD, in proportion to severity. [^11^C]UCB‐J PET could support translational studies and experimental medicine strategies for new disease‐modifying treatments for neurodegeneration. ANN NEUROL 2023;93:142–154

## Introduction

Frontotemporal dementia is a clinically and pathologically heterogeneous group of neurodegenerative conditions. Similar clinical phenotypes arise from different pathologies,^1^, ^2^ including 3R or 4R tauopathy, and TDP‐43 pathology. Despite this molecular heterogeneity, postmortem human studies^3^, ^4^, ^5^ and animal models^6^ suggest that the spectrum of frontotemporal dementia is characterized by early and severe synaptic loss, even preceding neuronal death and atrophy.

Our hypothesis was that in people with frontotemporal dementia, synaptic loss is severe and proportionate to clinical severity. Regionally specific and clinically relevant loss of synapses has been shown in the tauopathies of Alzheimer disease,^7^, ^8^, ^9^, ^10^, ^11^ progressive supranuclear palsy, and corticobasal degeneration.^12^, ^13^ TDP‐43‐related frontotemporal lobar degeneration is also associated with synaptic loss.^14^ For the behavioral variant of frontotemporal dementia (bvFTD), characterized by progressive personality and behavior changes, and cognitive decline, including executive impairments,^15^ we propose that synaptic loss would be most prominent in frontotemporal regions.

Previous evidence for synaptic loss in frontotemporal dementia has been indirect, including reduced synaptic density in carriers of mutations associated with frontotemporal dementia,^16^ abnormal synaptic markers in cerebrospinal fluid (CSF),^17^, ^18^ and progressive frontotemporal hypometabolism that is disproportionate to atrophy, indicated by [^18^F]FDG positron emission tomography (PET)^19^ (see Chételat et al^20^ for review). Recently, new tools to quantify synaptic density in vivo have been developed, including radioligands that bind selectively to synaptic vesicle protein 2A (SV2A) as an assay of synaptic density.^21^ Synaptic density changes only slightly in mid and later life,^22^, ^23^ providing a stable background against which to assess the effect of disease and disease severity.

Here, we used PET with the SV2A radioligand [^11^C]UCB‐J to assess the regional distribution of synaptic loss in patients with a clinical diagnosis of probable bvFTD, compared to age‐ and sex‐matched controls. Synaptic density was estimated using nondisplaceable binding potential (BP_ND_), a metric proportional to binding site density. In this context, if proven useful, [11C]UCB‐J PET could become an important tool for patient stratification and a surrogate endpoint in clinical trials and early interventions. PET measures for synaptic density would give additive and complementary information to fluid marker evidence, which does not elucidate in vivo spatial distributions of brain changes and may be less sensitive in prodromal stages. These characteristics of [^11^C]UCB‐J therefore permit its use as an in vivo marker for synaptic loss in frontotemporal dementia, irrespective of whether the underlying pathology is tau or TDP‐43. We predicted substantial synaptic loss in frontotemporal regions across all patients, and a correlation with clinical severity.

## Subjects and Methods

### Participants

Eleven patients with a clinical diagnosis of probable bvFTD^15^ were recruited from the Cambridge Centre for Frontotemporal Dementia at Cambridge University Hospitals National Health Service Foundation Trust and the University of Cambridge. Twenty‐five healthy volunteers were recruited from the UK National Institute for Health Research Join Dementia Research register.

Participants were screened for exclusion criteria: current or recent history (within the past 5 years) of cancer, current use of the anticonvulsant medication levetiracetam (which binds to SV2A, the target of [^11^C]UCB‐J), history or magnetic resonance imaging (MRI) evidence of ischemic or hemorrhagic stroke, any severe physical illness or comorbidity that would limit the ability to fully and safely participate in the study, and any contraindications to MRI. Eligible volunteers underwent a clinical and neuropsychological assessment including the Mini‐Mental State Examination (MMSE), revised Addenbrooke Cognitive Examination (ACE‐R), INECO frontal screening (IFS), Frontotemporal Dementia Rating Scale (FTD‐RS), and the Clinical Dementia Rating Scale (CDR). FTD‐RS was only acquired for patients, as it is a caregiver‐based assessment of patients' performance in activities of daily living. Within 3 months, all participants underwent brain imaging with 3T MRI and PET scanning with [^11^C]UCB‐J ((*R*)‐1‐((3‐(methyl‐^11^C)pyridin‐4‐yl)methyl)‐4‐(3,4,5‐trifluorophenyl)pyr‐rolidin‐2‐one).

The research protocol was approved by the Cambridge Research Ethics Committee (18/EE/0059) and the Administration of Radioactive Substances Advisory Committee. All participants provided written informed consent in accordance with the Declaration of Helsinki.

### Imaging Acquisition and Processing

Full details of the protocol for [^11^C]UCB‐J synthesis, data acquisition, image reconstruction, and kinetic analysis have been published elsewhere.^12^, ^16^ In brief, dynamic PET data acquisition was performed on a SIGNA PET/MR device (GE Healthcare, Waukesha, WI) for 90 minutes following [^11^C]UCB‐J injection, with attenuation correction including the use of a multisubject atlas method^24^ and improvements to the brain MRI coil component. Each emission image series was aligned and rigidly registered to T1‐weighted MRI acquired in the same session (repetition time = 3.6 milliseconds, echo time = 9.2 milliseconds, 192 sagittal slices, in‐plane voxel dimensions = 0.55 × 0.55mm [subsequently interpolated to 1.0 × 1.0mm], slice thickness = 1.0mm).

For each subject, a [^11^C]UCB‐J BP_ND_ map was determined from dynamic images corrected for partial volume effects at the voxel level using the iterative Yang method.^25^ BP_ND_ was calculated using a basis function implementation of the simplified reference tissue model, with centrum semiovale as the reference tissue.^26^ The reference region in the centrum semiovale was delineated using a lower threshold of 98% on the SPM12 white matter probability map smoothed to match the PET spatial resolution. Each BP_ND_ map was warped to the ICBM 152 2009a asymmetric magnetic resonance (MR) template using parameters from the spatial normalization of the coregistered T1 MR image with Advanced Normalization Tools (http://www.picsl.upenn.edu/ANTS/). For voxelwise analyses, the spatially normalized [^11^C]UCB‐J BP_ND_ maps were spatially smoothed with a full width at half maximum (FWHM) Gaussian kernel to an effective estimated smoothness of 10mm, prior to statistical analysis. T1‐weighted MRIs were segmented using the Computational Anatomy Toolbox 12 (http://www.neuro.uni-jena.de/cat/), and total intracranial volume (TIV) was estimated as the sum of gray matter, white matter, and CSF segmentations. Gray matter maps were warped to Montreal Neurological Institute space and smoothed with a 10mm FWHM Gaussian kernel prior to statistical analysis.

For regional analysis, we used the n30r83 Hammers atlas (http://brain-development.org) modified to include segmentation of brainstem and cerebellum, and nonrigidly registered to the T1‐weighted MRI of each participant. Regions were multiplied by a binary gray matter mask denoting >50% on the gray matter tissue probability map (SPM12 v7771, Institute of Neurology, London, UK) smoothed to PET spatial resolution, and geometric transfer matrix (GTM) partial volume correction was applied to each image of the dynamic series. Regional BP_ND_ was determined with the same kinetic modeling approach and reference tissue as for the BP_ND_ maps. Gray matter volumes for Hammers regions multiplied by the gray matter mask were also extracted.

### Statistical Analyses

Voxelwise analyses were performed using SPM12. First, a 2‐sample *t* test was performed to compare the bvFTD group to controls across the whole brain. Second, to test the association between synaptic density and clinical severity in people with bvFTD, voxelwise general linear models were applied across all patients, including ACE‐R and IFS scores as dependent variables in separate models (for IFS, the analyses used 10 patients due to missing data). Similar models were applied to test for associations between [^11^C]UCB‐J BP_ND_, age, and symptom duration. All results were tested at an uncorrected voxel height threshold of *p* < 0.001 combined with a familywise error (FWE) corrected cluster threshold of *p* < 0.05; peak voxels in clusters that reached the conservative voxel‐level FWE threshold (FWE *p* < 0.05) are also indicated.

We performed complementary analyses on regions of interest, using R version 4.0.0 (R Core team 2020, https://www.r-project.org/) and JASP (JASP team, https://jasp-stats.org/). [^11^C]UCB‐J BP_ND_ values from regions of interest were aggregated into left and right frontal lobe, temporal lobe, parietal lobe, occipital lobe, and cingulate cortex, and included in the analyses alongside insular cortex, hippocampus, amygdala, and thalamus. First, 2‐sample *t* tests were performed in each region to compare the bvFTD group to controls. Across all brain regions, *p* values from *t* tests were corrected with false discovery rate correction, and Cohen *d* effect sizes were calculated. As explorative analyses, we also performed 2‐sample *t* tests on [^11^C]UCB‐J BP_ND_ values of bvFTD group versus controls considering smaller regions, obtained from the Hammers atlas with and without partial volume correction. Second, Spearman correlation analyses were performed to test the association between cognitive performance and [^11^C]UCB‐J BP_ND_ in cortical regions. In addition to the above frequentist statistics, we also present Bayesian analogous tests for regional effects (https://osf.io/gny35/), complementing the strength of the evidence for the null versus alternate hypotheses.

Next, we compared the severity of synaptic loss (PET) and atrophy (MRI). The analysis of gray matter volume loss, at voxelwise and regional levels, was analogous to the [^11^C]UCB‐J PET analyses. First, we ran voxelwise group comparison analyses (controls vs patients), using 10mm‐smoothed gray matter images, including TIV as covariate (or scaling factor). Second, we considered regional partial volume corrected [^11^C]UCB‐J BP_ND_ and gray matter volumes (divided by TIV) extracted using the Hammers atlas. We calculated regional *z* scores for each patient and modality using regional means and standard deviations from the controls. We compared regional *z* scores between the two modalities, identifying regions where patient‐average *z* score values for [^11^C]UCB‐J BP_ND_ were lower than *z* scores for gray matter volumes. We ran a further explorative analysis to assess the association between regional gray matter *z* scores and regional [^11^C]UCB‐J BP_ND_ *z* scores (determined from BP_ND_ values without partial volume correction), using a linear mixed effects model (lmer function in R) with uncorrelated random intercept and slope terms, to take into account the variability between subjects in relation to the modalities (see Malpetti et al^27^ for details on the approach).

Finally, to test whether the apparent effect of bvFTD on synaptic loss (quantified with [^11^C]UCB‐J PET) is attributable to the associated gray matter atrophy, we ran 2 additional analyses. First, we ran region‐specific logistic regression models, with group as the dependent variable, and either gray matter volumes as the predictor or both gray matter volumes and [^11^C]UCB‐J binding potential. The first model tests whether individuals can be classed as patient versus control based on their atrophy alone, whereas the second considers the additional effect of synaptic density. We compared the models by analysis of variance and Akaike information criterion. Second, we repeated the regional group comparison analyses for [^11^C]UCB‐J PET after regressing out the region‐specific gray matter volumes. We used partial volume noncorrected binding potential as the dependent variable and gray matter volume as the predictor in a linear regression model and took the resulting residuals to *t* test between groups.

## Results

### Descriptive Statistics

Demographic and clinical features for the two groups are given in Table 1. The groups were similar in age and sex, with cognitive deficits among patients typical for bvFTD. The bvFTD group showed reduced performance in all cognitive domains. Performance on visuospatial tasks was also impaired, but this may reflect language or executive impairment over and above true visuospatial deficits, which were not prominent in any of the patients' clinical history. The mean FTD‐RS logit of −2.6 lies at the boundary between moderate and severe impairment.

**TABLE 1:** Demographic and Clinical Characteristics for Control and bvFTD Groups

Characteristic	Controls, n = 25	bvFTD, n = 11	Difference
Age, yr	70.2 (7.0)	65.7 (9.3)	n.s.
Sex, F:M	9:16	2:9	n.s.
MMSE, /30	29.5 (1.1)	22.3 (8.6)	*t*(10.1) = 2.8, *p* = 0.019, *d* = 1.2
ACE‐R, /100	96.2 (2.5)	63.0 (26.9)	*t*(10.1) = 4.1, *p* = 0.002, *d* = 1.7
Att/orient, /18	17.9 (0.3)	13.0 (5.6)	*t*(10.0) = 2.9, *p* = 0.016, *d* = 1.2
Memory, /26	24.7 (1.7)	14.5 (8.4)	*t*(10.3) = 4.0, *p* = 0.002, *d* = 1.7
Fluency, /14	12.4 (1.5)	4.4 (4.5)	*t*(10.0) = 5.8, *p* < 0.001, *d* = 2.4
Language, /26	25.6 (0.8)	19.1 (7.6)	*t*(10.1) = 2.8, *p* = 0.018, *d* = 1.2
Visuospatial, /16	15.6 (0.6)	12.1 (4.0)	*t*(10.2) = 2.9, *p* = 0.015, *d* = 1.2
IFS	25.7 (2.4)	9.4 (7.1)	*t*(9.9) = 7.0, *p* < 0.001, *d* = 3.1
IFS WM	7.6 (1.3)	3.7 (2.4)	*t*(11.4) = 5.0, *p* < 0.001, *d* = 2.1
Symptom duration, mo	—	79.9 (40.6)	—
FTD‐RS, %	—	18.9 (18.9)	—
FTD‐RS, logit	—	−2.6 (1.7)	—

*Note*: Mean and standard deviation are reported for each continuous variable. Group comparisons were performed with 2‐sample *t* tests for continuous variables, and chi‐squared test for sex.

Abbreviation: ACE‐R = revised Addenbrooke Cognitive Examination; Att/orient = attention/orientation score; bvFTD = behavioral variant of frontotemporal dementia; *d* = Cohen *d*; F = female; FTD‐RS = Frontotemporal Dementia Rating Scale; IFS = INECO frontal screening; M = male; MMSE = Mini‐Mental State Examination; n.s. = not significant; WM = working memory.

### Voxelwise [ ^11^C]UCB‐J Group Comparisons and Correlations with Cognitive Scores

**FIGURE 1:**
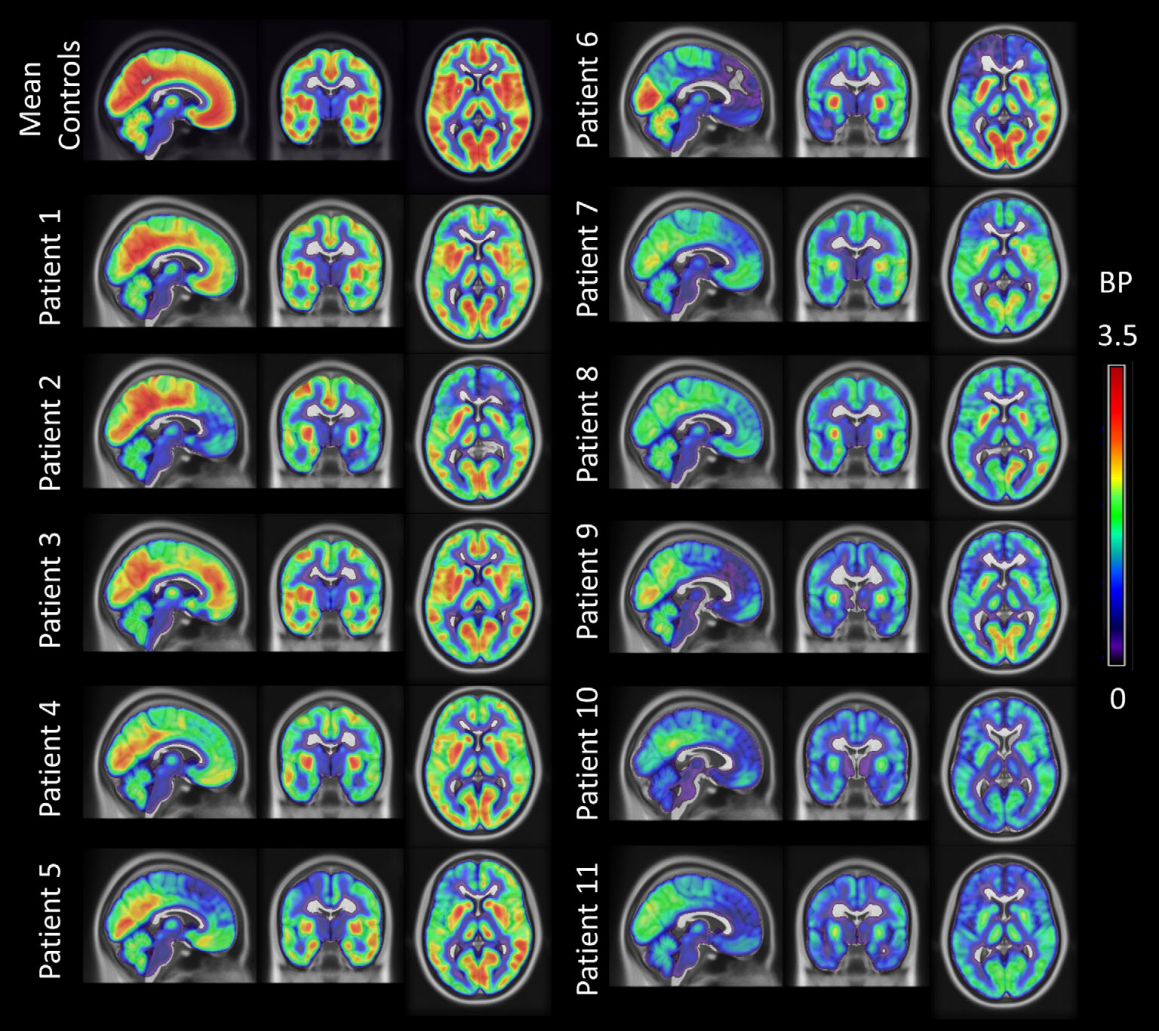
[^11^C]UCB‐J nondisplaceable binding potential (BP_ND_) maps for each behavioral variant of frontotemporal dementia patient. The BP_ND_ maps were spatially normalized to International Consortium for Brain Mapping (ICBM) 1522009a space, masked and smoothed (isotropic 6mm full width at half maximum Gaussian for visualization). The BP_ND_ maps are overlaid on the ICBM 1522009a T1 magnetic resonance template, and the slices are reported in the neurological display convention (left on the left). For comparison, the first row on the left shows the corresponding average BP_ND_ map across 25 controls.

Single‐subject [^11^C]UCB‐J BP_ND_ maps for individual patients and the average BP_ND_ map across all controls are reported in Figure 1, where higher [^11^C]UCB‐J BP_ND_ values (orange/red colors) represent higher synaptic density, and lower values (blue/green areas) represent lower synaptic density. People with bvFTD showed mild (Patient 1) to severe (Patient 11) regional synaptic loss compared to controls.

**FIGURE 2:**
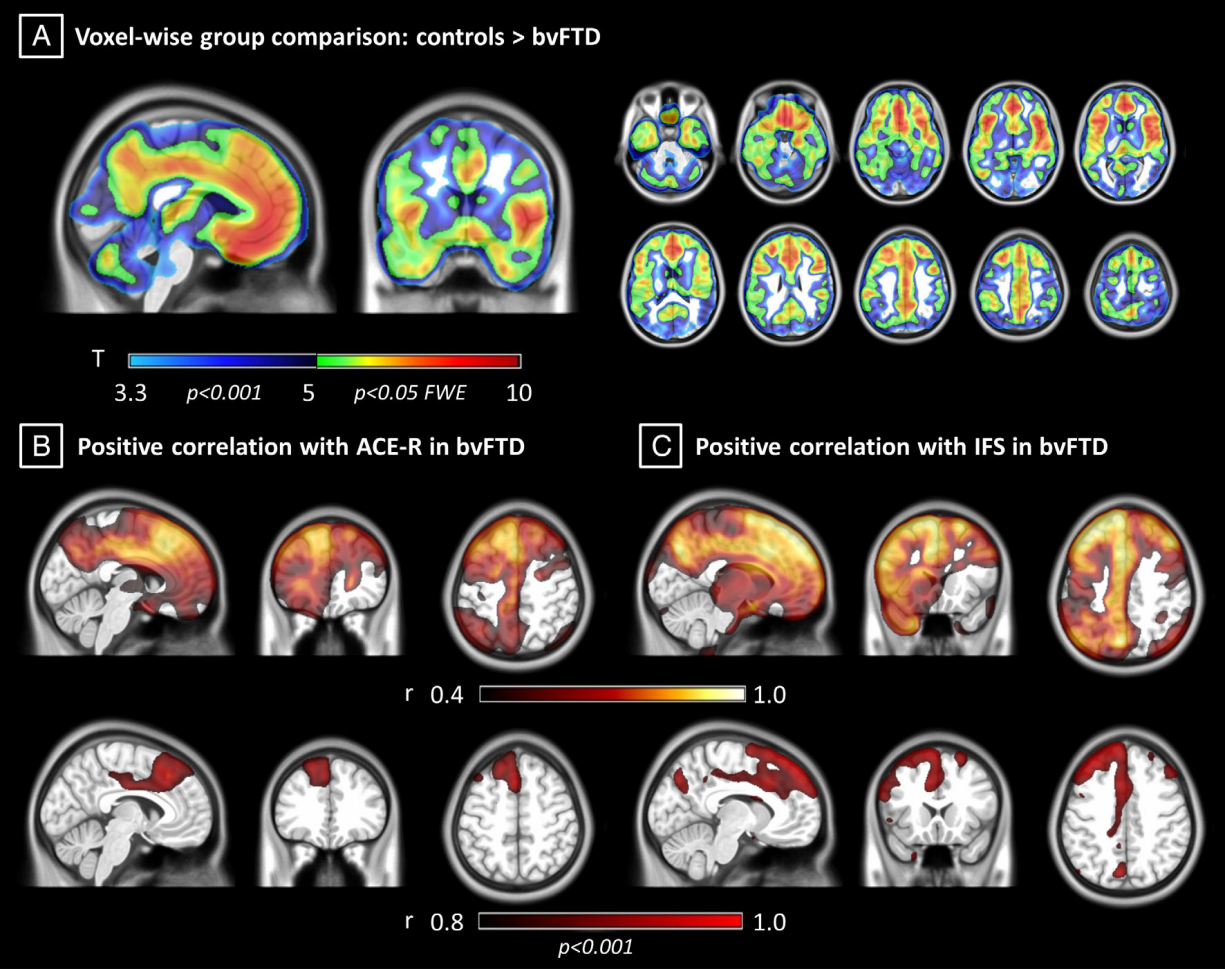
Voxelwise synaptic loss and association with cognitive impairments in behavioral variant of frontotemporal dementia (bvFTD). (A) T maps from voxelwise analysis showing higher [^11^C]UCB‐J binding potential in controls compared to bvFTD (*p* < 0.05 familywise error [FWE] corrected at voxel level). (B, C) Coefficient of correlation maps for the bvFTD group between voxelwise [^11^C]UCB‐J binding potential and cognitive performance on revised Addenbrooke Cognitive Examination (ACE‐R) and INECO frontal screening (IFS; *p* < 0.001 uncorrected at voxel level, *p* < 0.05 FWE corrected at cluster level).

At the FWE corrected voxel‐level threshold (*p* < 0.05), the 2‐sample *t* test on [^11^C]UCB‐J BP_ND_ maps revealed synaptic loss in the bvFTD group compared to controls (5.1 ≤ *t* ≤ 9.3) in frontal, temporal, insular, and cingulate cortex bilaterally (Fig 2A). Group differences were most pronounced in medial frontal and orbitofrontal cortex, superior frontal regions, inferior frontal gyrus, insular cortex, anterior and superior temporal lobe, and anterior cingulate cortex (*t* ≥ 6.4). The bvFTD group did not show significantly higher binding than controls in any area (*p* < 0.001 uncorrected).

For voxelwise correlation analyses between [^11^C]UCB‐J BP_ND_ and cognition, ACE‐R showed a strong positive association (*r* ≥ 0.8; 6.3 ≤ *t* ≤ 6.8, *p* < 0.001 uncorrected at the voxel level and *p* < 0.05 FWE corrected at the cluster level) between cognition and [^11^C]UCB‐J BP_ND_ in the anterior cingulate gyrus bilaterally, the left superior and middle frontal gyri, and the medial anterior temporal lobe (see Fig 2B). IFS scores correlated positively (*r* ≥ 0.8; 7.9 ≤ *t* ≤ 9.9, *p* < 0.001 uncorrected at voxel level and *p* < 0.05 FWE corrected at cluster level) with [^11^C]UCB‐J BP_ND_ in the superior and middle frontal gyri bilaterally, left cingulate cortex and inferior frontal gyrus, and left superior parietal gyrus and anterior medial temporal lobe (see Fig 2C). No significant negative correlations were found between [^11^C]UCB‐J BP_ND_ and either ACE‐R or IFS in any region (*p* < 0.001). Voxelwise correlation analyses did not reveal significant associations between [^11^C]UCB‐J BP_ND_, age, and symptom duration (*p* < 0.001 uncorrected).

### Region of Interest‐Based [ ^11^C]UCB‐J Group Comparisons and Correlations with Cognitive Scores

**FIGURE 3:**
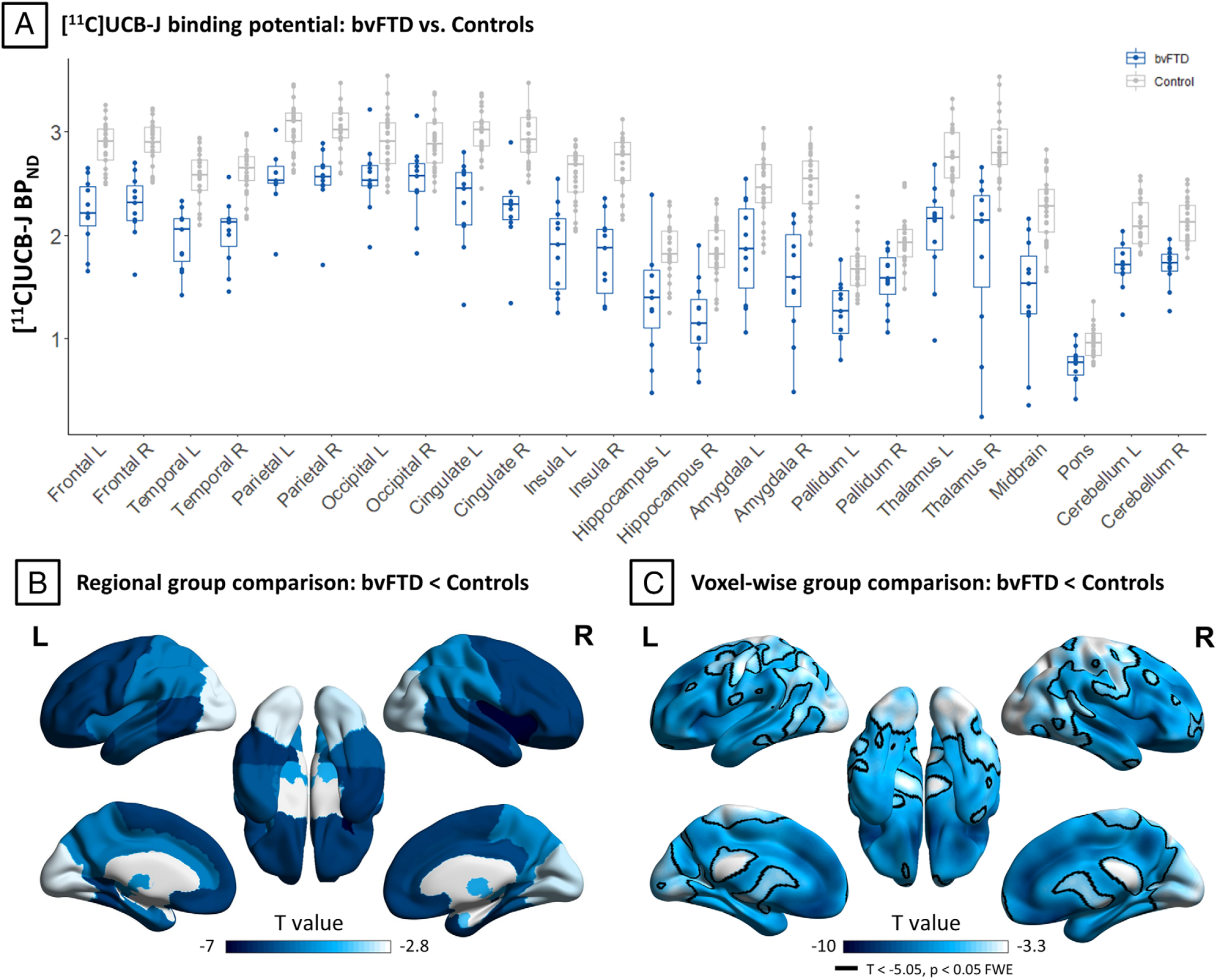
Regional partial volume corrected nondisplaceable binding potential (BP_ND_) values and group comparisons. (A) Regional [^11^C]UCB‐J BP_ND_ values for behavioral variant of frontotemporal dementia (bvFTD) patients (blue) and controls (gray). (B) Surface rendered regional *t* values for comparison of [^11^C]UCB‐J BP_ND_ between bvFTD patients and controls (*p* < 0.05 false discovery rate corrected). (C) For visual comparison with B, corresponding *t* values obtained from voxelwise group comparison. L = left; R = right.

Group comparison results by 2‐sample *t* tests in each region of interest are shown in Figure 3 and in more detail in Table 2. bvFTD caused severe synaptic loss in frontal and temporal lobes bilaterally, with the right cingulate and insular cortex showing the most severe loss (−6.9 ≤ *t* ≤ −5.6; −2.7 ≤ Cohen *d* ≤ −2.2). Results from explorative 2‐sample *t* tests on subregions are reported in the Supplementary Material, including partial volume corrected (Supplementary Table 1) and uncorrected (Supplementary Table 2) regional BP_ND_.

**TABLE 2:** Group Comparisons of Regional [^11^C]UCB‐J BP_ND_ with Partial Volume Correction Including Aggregated Regions [Color table can be viewed at www.annalsofneurology.org]

	BP_ND_				Statistical parameters			
	Mean CT	SD CT	Mean PT	SD PT	*t* value	Cohen's *d*	*p*	*p* FDR
Frontal L	2.87	0.22	2.22	0.33	−5.99	−2.33	0.0000	0.0001
Frontal R	2.90	0.21	2.28	0.31	−6.03	−2.33	0.0000	0.0001
Temporal L	2.57	0.24	1.96	0.30	−6.08	−2.28	0.0000	0.0001
Temporal R	2.62	0.22	2.03	0.32	−5.63	−2.17	0.0001	0.0002
Parietal L	3.04	0.24	2.54	0.29	−5.03	−1.89	0.0001	0.0003
Parietal R	3.01	0.22	2.53	0.31	−4.71	−1.81	0.0003	0.0004
Occipital L	2.90	0.28	2.56	0.33	−3.06	−1.14	0.0071	0.0075
Occipital R	2.89	0.25	2.51	0.35	−3.19	−1.23	0.0063	0.0071
Cingulate L	2.99	0.22	2.31	0.43	−4.98	−2.00	0.0003	0.0004
Cingulate R	2.94	0.24	2.24	0.37	−5.80	−2.26	0.0000	0.0002
Insula L	2.58	0.27	1.87	0.43	−5.13	−2.00	0.0002	0.0003
Insula R	2.69	0.26	1.79	0.39	−6.91	−2.68	0.0000	0.0001
Hippocampus L	1.84	0.27	1.36	0.53	−2.84	−1.14	0.0147	0.0147
Hippocampus R	1.84	0.28	1.17	0.39	−5.12	−1.96	0.0001	0.0003
Amygdala L	2.46	0.32	1.86	0.49	−3.80	−1.48	0.0020	0.0024
Amygdala R	2.50	0.30	1.57	0.55	−5.26	−2.09	0.0002	0.0003
Thalamus L	2.74	0.28	2.03	0.48	−4.57	−1.80	0.0005	0.0007
Thalamus R	2.86	0.31	1.86	0.79	−4.07	−1.67	0.0017	0.0022

Abbreviation: BP_ND_ = nondisplaceable binding potential; CT = controls; FDR = false discovery rate correction; L = left; PT = behavioral variant of frontotemporal dementia patients; R = right; SD = standard deviation.

*p* < 0.05 are highlighted in yellow, while *p* < 0.001 are highlighted in red.

**FIGURE 4:**
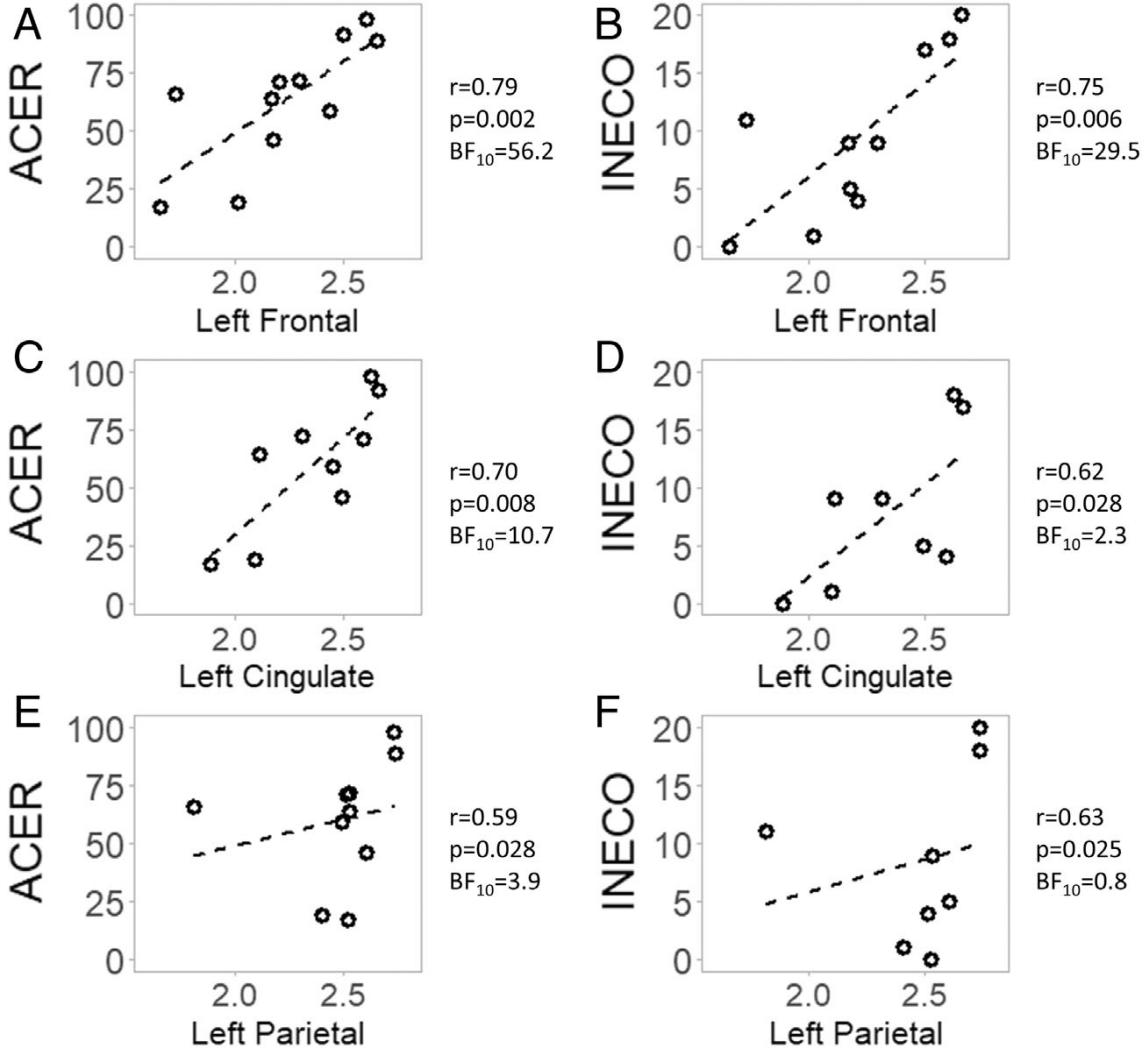
Correlations between regional synaptic density and cognitive performance. [^11^C]UCB‐J nondisplaceable binding potential (BP_ND_) in a cortical region is reported on the x axis, while the y axis represents a cognitive test score. ACER = revised Addenbrooke Cognitive Examination; BF = Bayes factor; *r* = Spearman rho.

Considering cortical regions of interest, Spearman correlation between partial volume corrected [^11^C]UCB‐J BP_ND_ and ACE‐R scores revealed strong positive associations in the left frontal lobe (*r* = 0.791, *p* = 0.002), cingulate cortex (*r* = 0.700, *p* = 0.008), and parietal lobe (*r* = 0.591, *p* = 0.028; Fig 4). Spearman correlation analyses with IFS scores showed positive associations with [^11^C]UCB‐J BP_ND_ in the same regions: the left frontal lobe (*r* = 0.754, *p* = 0.006), cingulate cortex (*r* = 0.620, *p* = 0.028), and parietal lobe (*r* = 0.632, *p* = 0.025). Similar results were obtained with regional BP_ND_ values without partial volume correction. Bayesian correlation analyses indicated strong evidence for the associations between [^11^C]UCB‐J BP_ND_ and ACE‐R scores in the left frontal lobe (Bayes factor [BF] = 56.2) and cingulate cortex (BF = 10.7), with positive evidence in the parietal lobe (BF = 3.9). Bayesian correlation analyses of IFS indicated strong evidence for positive correlation in the left frontal lobe (BF = 23.4) but a lack of positive evidence for or against a correlation in the left cingulate cortex (BF = 2.3) and parietal lobe (BF = 0.8). In Supplementary Table 3, explorative frequentist and Bayesian correlation analyses of regional [^11^C]UCB‐J BP_ND_ with MMSE, ACE‐R, and IFS subscores, and FTD‐RS are reported.

### Comparing [ ^11^C]UCB‐J Binding and Gray Matter Volume Loss in bvFTD

**FIGURE 5:**
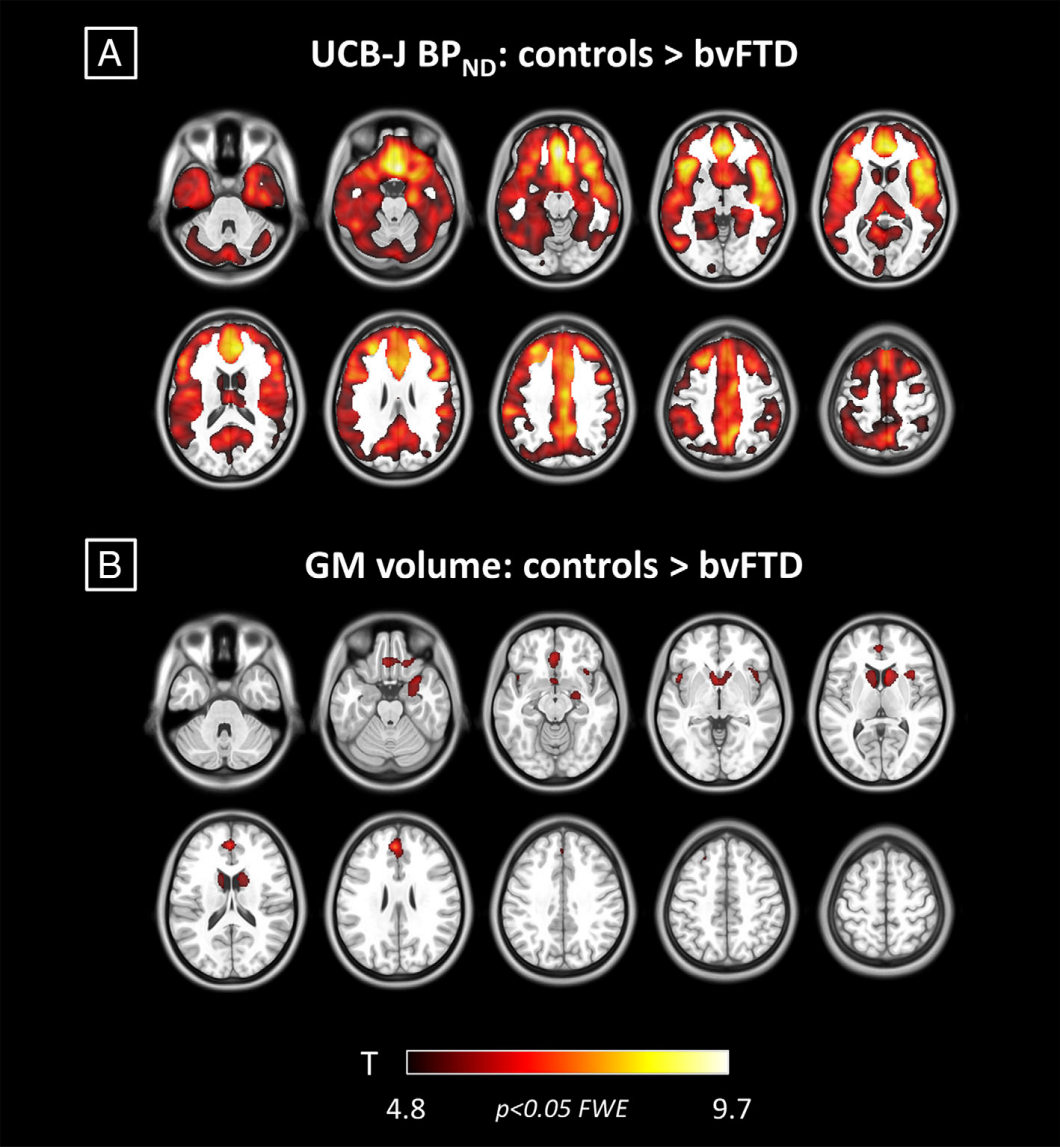
Synaptic loss and atrophy in patients with behavioral variant of frontotemporal dementia (bvFTD; *p* < 0.05 familywise error [FWE] corrected at voxel level). (A) T maps from voxelwise analysis showing higher [^11^C]UCB‐J nondisplaceable binding potential (BP_ND_) in controls compared to bvFTD. (B) T maps from voxelwise analysis showing higher gray matter (GM) volumes in controls compared to bvFTD. The color scale applies to both panels, showing colors associated to *t* values.

**FIGURE 6:**
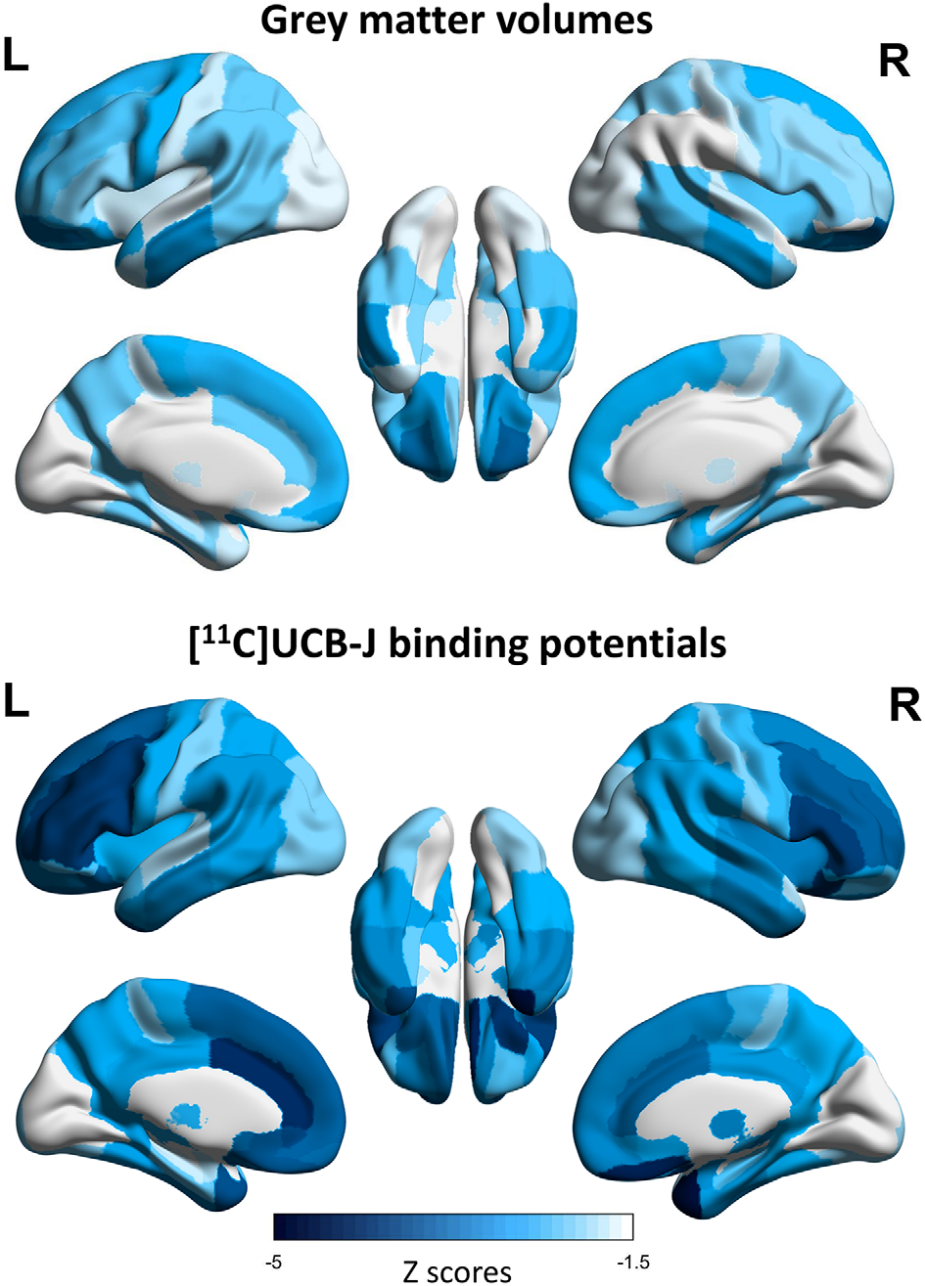
Regional gray matter volume *z* scores (top) and partial volume corrected [^11^C]UCB‐J binding potential *z* scores (bottom). For each modality, regional values represent average *z* scores across patients calculated using modality‐specific means and standard deviations of healthy controls. Darker colors represent greater negative regional *z* scores.

Voxelwise group comparisons with [^11^C]UCB‐J BP_ND_ and gray matter volumes show an extensive pattern of synaptic loss, and a more restricted pattern of gray matter atrophy in patients compared to controls (Fig 5 for FWE corrected results, and Supplementary Fig 1 for uncorrected *p* < 0.001). The regions of significant difference from both comparisons include medial frontal and orbitofrontal cortex, anterior cingulate gyrus, insula, anterior and medial temporal lobe, and striatum. Comparing regional *z* scores between the two modalities, we found that 84% of the regions have larger negative [^11^C]UCB‐J BP_ND_ effect sizes than gray matter volume effect sizes (Fig 6, Supplementary Table 4). In the analyses with uncorrected [^11^C]UCB‐J BP_ND_ regional values, ~95% of regions had larger negative effect sizes for [^11^C]UCB‐J BP_ND_ *z* scores than gray matter volume *z* scores (Supplementary Fig 2, Supplementary Table 4). A positive relationship between uncorrected [^11^C]UCB‐J BP_ND_ *z* scores and gray matter volume *z* scores across all regions was confirmed at the group level (estimate = 0.41, standard error = 0.08, *p* = 0.0004). The strength of this association (slope) varied between patients, but was positive in all cases (Supplementary Fig 3).

The results of logistic regression analyses are described in Supplementary Table 5. The model comparisons suggested that for all regions (except caudate), adding regional [^11^C]UCB‐J values to the model led to a significantly improved fit (over the model with structural MRI as the only predictor), even after penalization for increased model complexity. Group comparison of regional residuals of uncorrected [^11^C]UCB‐J BP_ND_ after regressing out region‐specific gray matter volumes is described in Supplementary Table 6. Most brain regions still showed significant reductions in patients versus controls, and none showed the reverse effect.

## Discussion

This study confirms the hypothesis that people with a clinical diagnosis of bvFTD have severe and widespread synaptic loss over frontotemporal cortex, including cingulum and insula. The synaptic density in frontal and cingulate regions as quantified with [^11^C]UCB‐J BP_ND_ correlates with patients' cognitive performance. [^11^C]UCB‐J BP_ND_ was not related to age, in line with previous evidence.^23^ These principal results were observed with and without partial volume correction, and using both voxelwise and regional analysis. Of note, the effect size and regional extent of significant synaptic loss were greater than the effect size and spatial extent of significant gray matter atrophy, as detected by T1‐weighted MRI. The loss of synapses detected by [^11^C]UCB‐J PET raises 3 points of particular importance.

First, the decline of synaptic health—including synaptic density and synaptic plasticity—will directly impact brain connectivity and learning. Synaptic mediation of neurophysiological connectivity underpins cognitive function, and synaptic plasticity is one of the major determinants of learning and memory in multiple cognitive systems. In Alzheimer disease, synaptic loss is more closely related to cognitive impairment than tau burden, beta‐amyloid burden, or neuronal loss.^28^ Such a preeminent relationship may also hold in frontotemporal dementia. Here, we observed bilateral synaptic loss in patients, but the associations between synaptic density and cognitive performance were left‐lateralized. This might reflect asymmetric synaptic loss in some patients (eg, Patients 6 and 7 in Fig 1), but also the strong dependency of ACE‐R and IFS cognitive assessments on language. The distinctive distribution of synaptic loss in bvFTD across frontotemporal, insular, and cingulate cortex is consistent with the typical distribution of its molecular pathologies, and its neurocognitive and behavioral profile. In this study, all of our cases were symptomatic (CDR ≥ 1), although we note that behavioral and cognitive changes can emerge in the presymptomatic and prodromal stages of bvFTD, many years before dementia and diagnosis^29^, ^30^; and synaptic loss has been identified in the presymptomatic stage of those with highly penetrant mutations such as C9orf72 expansions.^16^ Early synaptic dysfunction in these regions may explain the subtle presymptomatic behavioral change and executive dysfunction. In addition, synaptic loss may determine the neurophysiological signatures of bvFTD as assayed by magnetoencephalography as in Alzheimer disease.^9^

Second, quantifying the degree and distribution of synaptic loss can enrich models of frontotemporal dementia pathogenesis, in preclinical and clinical settings. For example, in preclinical tauopathy models, connectivity more than proximity has been shown to influence the spread of diverse toxic oligomeric tau species.^31^ Several mechanisms have been demonstrated at the synapse by which such toxic oligomeric tau can be transferred between neurons. The oligomeric tau is in turn synaptotoxic,^32^ and affects synaptic health even in the absence of cell death. In Alzheimer disease, in vivo human PET and CSF studies recently demonstrated a strong association between tau pathology and reduced synaptic integrity.^9^, ^10^, ^33^ In other clinical disorders caused by tau‐mediated frontotemporal lobar degeneration, the tauopathy seems to be synaptotoxic,^13^ whereas synaptic density and connectivity seem to confer vulnerability to molecular pathology.^13^, ^34^ In preclinical models, [^11^C]UCB‐J may now be used in autoradiography or in vivo PET^22^, ^35^, ^36^, ^37^ to quantify synapse density, where it is highly correlated with better established markers such as synaptophysin.^22^ [^11^C]UCB‐J therefore offers a bridge between preclinical and clinical models, where quantitative whole brain models of pathogenesis in dementia, including frontotemporal dementia, can be improved by the inclusion of synaptic density estimates in addition to structural connectivity^38^ and postsynaptic structural integrity.^39^ In addition, it is possible that tau and TDP‐43‐mediated bvFTD have distinct spatiotemporal profiles of synaptic loss. This could be tested with studies using [^11^C]UCB‐J PET in preclinical models and people with genetically determined familial frontotemporal dementia,^17^, ^18^ or in due course by postmortem examination of those who have undergone PET. However, we suggest that synaptic loss is a mechanistic convergence point, present across diverse molecular pathologies.

Third, the ability to quantify the degree and distribution of synaptic loss can enhance novel experimental medicine studies,^40^ given the critical role synaptic health plays in mediating between the molecular pathology and cognitive physiological impairment in frontotemporal dementia. The degree and distribution of loss may be used either for inclusion stratification or for analytical stages of an early phase clinical trial. It may also be considered as an intermediate marker of efficacy, particularly where the mechanisms of action of a drug are upstream of synaptic loss or are directly related to synaptic resilience and preservation.

[^11^C]UCB‐J is not the only potential marker of synaptic health in frontotemporal dementia and related disorders. A previous study using [^18^F]UCB‐H PET to assess synaptic loss did not identify a deficit in frontotemporal dementia versus controls, nor was there a difference between frontotemporal dementia and Alzheimer's disease.^41^ Compared to Salmon et al,^41^ our cohort of bvFTD patients had longer symptom duration (6.7 vs 5.2 years), lower cognitive performance (eg, MMSE 22.3 vs 25.4), and younger age (65.7 vs 73.5 years). The lower specific binding of [^18^F]UCB‐H compared to [^11^C]UCB‐J may also have contributed to their null result. Furthermore, blood‐based kinetic analysis was used to determine total volume of distribution (V_T_) for [^18^F]UCB‐H, which includes nonspecific binding and free tracer in tissue. Hence, [^18^F]UCB‐H V_T_ may be less sensitive to synaptic changes than [^11^C]UCB‐J BP_ND_. Results with the alternative ligand [^18^F]SDM8/SynVesT‐1 have not yet been reported in frontotemporal dementia. Several studies have used [^18^F]FDG PET to assess brain metabolism and interpreted changes as a marker of synaptic loss. Such metabolic change is disproportionate to atrophy,^19^ but is not a direct measure of synaptic density. The direct comparison between [^11^C]UCB‐J and [^18^F]FDG PET in Alzheimer's disease indicated a high correlation in medial temporal regions, but not elsewhere^42^; measures of synaptic loss and hypometabolism may therefore provide complementary information about the underlying pathophysiology. In addition to [^18^F]fluorodeoxyglucose and [^11^C]UCB‐J PET, synaptic function may be assessed by electro−/magnetoencephalography. For example, in Alzheimer's disease, tau burden ([^18^F]flortaucipir) and synaptic loss ([^11^C]UCB‐J) are both associated with changes in magnetoencephalography.^9^ Fluidic markers of synaptic health are also emerging; among these, growth‐associated protein 43, synaptosomal‐associated protein 25, and synaptotagmin‐1 are the main presynaptic markers that have been applied in Alzheimer disease, followed more recently by proteomic studies that found decreased neurofascin, neuronal pentraxin 1, and neurexin 1 in Alzheimer disease (see Camporesi et al^43^ for a review). In frontotemporal dementia, fewer markers for synaptic health have been identified as useful, including pentraxins.^17^, ^44^ In particular, neuronal pentraxin 2 is described as the earliest biomarker to become detectably abnormal, as compared to other CSF markers, in presymptomatic carriers of gene mutations related to frontotemporal dementia.^44^ However, these markers are largely restricted to CSF‐based rather than blood‐based assays. In comparison to other techniques, [^11^C]UCB‐J PET has excellent reproducibility, which is advantageous for longitudinal and interventional studies.

Further studies are needed to develop the utility of synaptic PET as a clinical and/or research tool in frontotemporal dementia. However, our results suggest that it may be useful for the quantification and localization of a major etiopathogenic process such as synaptic loss, and cannot be reduced to cell loss, which can be assessed with structural MRI. Our direct comparison of voxelwise and regional analyses with [^11^C]UCB‐J binding potentials and gray matter volumes identified an extensive pattern of synaptic loss, and a more restricted pattern of gray matter loss in patients with bvFTD compared to controls. Specifically, the atrophy pattern in these patients was a subregion of the more widespread significant synaptic loss. Confirmatory analyses showed that regressing the effect of atrophy (using the residual approach or including both [^11^C]UCB‐J and structural MRI regional measures in the same model), [^11^C]UCB‐J PET leaves widespread significant differences in regional synaptic density between patients and controls. One could interpret [^11^C]UCB‐J PET as being more sensitive to the presence of brain changes associated with frontotemporal dementia, and conclude that detectable synaptic loss precedes detectable atrophy in patients with bvFTD.^3^, ^4^, ^5^, ^6^ The histopathological basis of the changes underlying MRI measures of atrophy are complex, but ultimately rest on large‐scale cell loss. The loss of synapses on dendrites, and dendritic dearborization, precede cell death. This observation of early synaptic loss before cell loss or atrophy is seen in preclinical models of tauopathy^45^ and in genetic mutation carriers at risk of frontotemporal dementia.^16^ In this context, [^11^C]UCB‐J PET has potential advantages over MRI for 3 main reasons. First, it allows in vivo quantification in humans of a process that in preclinical models, including transgenic animal models, has been shown to occur before neurodegeneration and cell death, and to occur in response to changes in proteostasis and inflammation. Second, it provides direct evidence for synaptic loss as one of the processes that is commonly presented in depictions of the hypothesis of cascading “multiple biomarkers,” which has to date lacked direct evidence in humans. Third, it provides a readout tool for experimental medicine studies that target processes upstream of synaptic loss.

Our study has several limitations. We acknowledge the relatively small sample size, which does not permit statistically elaborate models (ie, with multiple covariates, such as age and sex, which have previously been shown to have minor/negligible effects on synaptic PET signal^23^). However, the expected effect size in frontotemporal dementia was large (Cohen *d* > 1), and power was estimated to be sufficient (β > 0.8 for α < 0.05). Moreover, we found consensus across different analytic methods: voxelwise and ROI‐based analyses, and BP_ND_ values determined from data with and without partial volume correction. The Bayesian tests confirmed that we had sufficient precision (analogous to power in frequentist tests) to support the alternate hypotheses (BFs > 3) with strong evidence from the 11 patients' data. The convergence over these statistical approaches mitigates against inadequate power and sample‐dependent biases on the estimation of group differences and imaging–cognition associations. The replication of these findings with larger and multicenter clinical cohorts will nonetheless represent an important step to establish the generalizability of our results, and utility for clinical trials. Recruitment was based on clinical diagnosis, rather than neuropathology or genetics, but the clinical diagnosis was reconfirmed at serial clinical visits and has high clinicopathological correlation with either frontotemporal lobar degeneration (FTLD)–tau or FTLD–TDP‐43. Given the novelty of the radiotracer, the cohort has been scanned recently (within 24 months of submission), meaning survival analyses and neuropathological confirmation are not yet possible. Future PET‐to‐autopsy studies will be needed to investigate the association between in vivo measures of synaptic density and neuropathology. Partial volume correction methods (iterative Yang [voxelwise] and GTM [regional]) have been used to minimize the effect of atrophy on the [^11^C]UCB‐J binding potential group comparisons and correlations, and to assess the variance and effects explained by synaptic loss over and above atrophy. Synaptic loss shows a more extensive pattern than gray matter loss in frontotemporal dementia, and the concordance of partial volume corrected and noncorrected analyses already indicates that the synaptic loss we observe is not simply attributable to atrophy.

To conclude, our study confirms that bvFTD is associated with significant and widespread frontotemporal loss of synapses, in proportion to disease severity. We suggest that [^11^C]UCB‐J PET can facilitate the validation of preclinical models, inform models of human pathogenesis, and inform the design of new disease‐modifying treatment strategies.

## Author Contributions

M.M., J.T.O., and J.B.R. contributed to the conception and design of the study. M.M., P.S.J., T.E.C, N.H., M.N., M.A.R., T.R., G.S., D.J.W., D.S., T.D.F., Y.T.H., S.M.S., and F.I.A. contributed to the acquisition and analysis of data. M.M., J.T.O., and J.B.R. contributed to drafting the text or preparing the figures.

## Potential Conflicts of Interest

Nothing to report.

## Supporting information


**APPENDIX S1.** Supporting Information

## Data Availability

Anonymized data may be shared upon request to the corresponding or senior author from a qualified investigator for noncommercial use, subject to restrictions according to participant consent and data protection legislation.
