# Supplementary material for: Synaptic Loss in Frontotemporal Dementia Revealed by [ 11C]UCB‐J Positron Emission Tomography
Source: Ann Neurol. 2022 Nov 16;93(1):142–54. doi: 10.1002/ana.26543 (PMC10099663; doi:10.1002/ana.26543)
Supplement: Supplementary file 1 — APPENDIX S1. Supporting Information [file ANA-93-142-s001.docx]

**Supplementary Material**

**Synaptic loss in frontotemporal dementia revealed by [^11^C]UCB-J PET**

Maura Malpetti^1^, P. Simon Jones^1^, Thomas E. Cope^1,2,3^, Negin Holland^1,2^, Michelle Naessens^1^, Matthew A. Rouse^3^, Timothy Rittman^1,2^, George Savulich^5^, David J. Whiteside^1,2^, Duncan Street^1,2^, Tim D. Fryer^1,4^, Young T. Hong^1,4^, Selena Milicevic Sephton^1,4^, Franklin I. Aigbirhio^1,4^, John T. O’Brien^2,5#^, James B. Rowe^1,2,3#^

# Joint senior authors

**Author affiliations:**

1 Department of Clinical Neurosciences, University of Cambridge, Cambridge, UK

2 Cambridge University Hospitals NHS Foundation Trust, Cambridge, UK

3 Medical Research Council Cognition and Brain Sciences Unit, University of Cambridge, UK

4 Wolfson Brain Imaging Centre, University of Cambridge, Cambridge, UK

5 Department of Psychiatry, University of Cambridge, Cambridge, UK

**Corresponding Author:**

Dr. Maura Malpetti

Department of Clinical Neurosciences
University of Cambridge
Herchel Smith Building, Forvie Site
Robinson Way, Cambridge Biomedical Campus
Cambridge CB2 0SZ

Email: [mm2243@medschl.cam.ac.uk](mailto:mm2243@medschl.cam.ac.uk)

**Supplementary Figure 1. Synaptic loss and atrophy in patients with bvFTD.** Panel A: t maps from voxel-wise analysis showing higher [^11^C]UCB-J binding potential (BP_ND_) in controls compared to bvFTD. Panels B: t maps from voxel-wise analysis showing higher grey-matter (GM) volumes in controls compared to bvFTD. The colour scale applies to both panels, showing colours associated to t values (T val) and respective p values (p < 0.001 uncorrected, p < 0.05 FWE-corrected at voxel level).

**
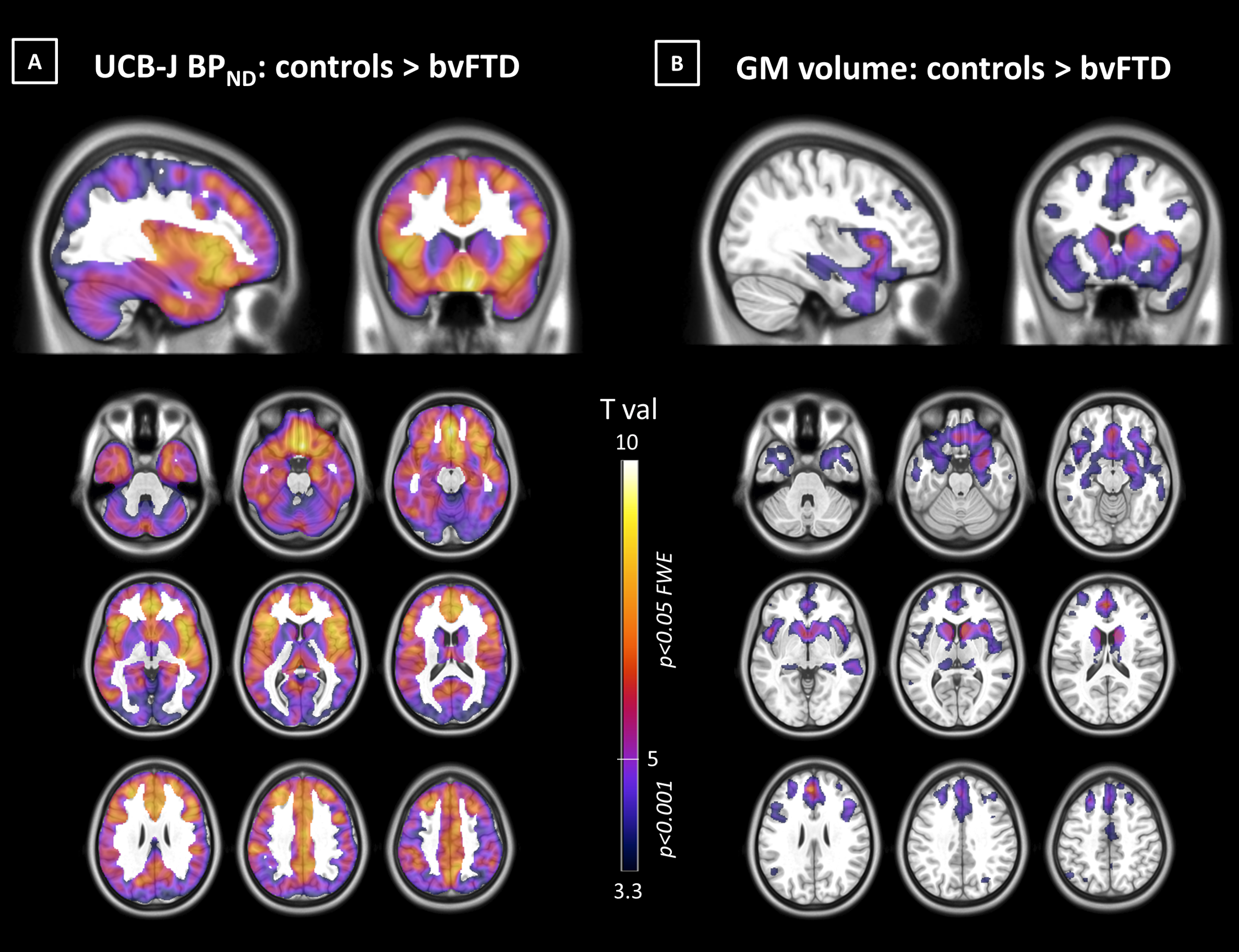
**

**Supplementary Figure 2. Regional partial-volume corrected (top) and uncorrected (bottom) [^11^C]UCB-J binding potential z-scores.** Regional values represent average z-scores across patients calculated using modality-specific means and standard deviations of healthy controls. Darker colours represent greater negative regional z-scores.


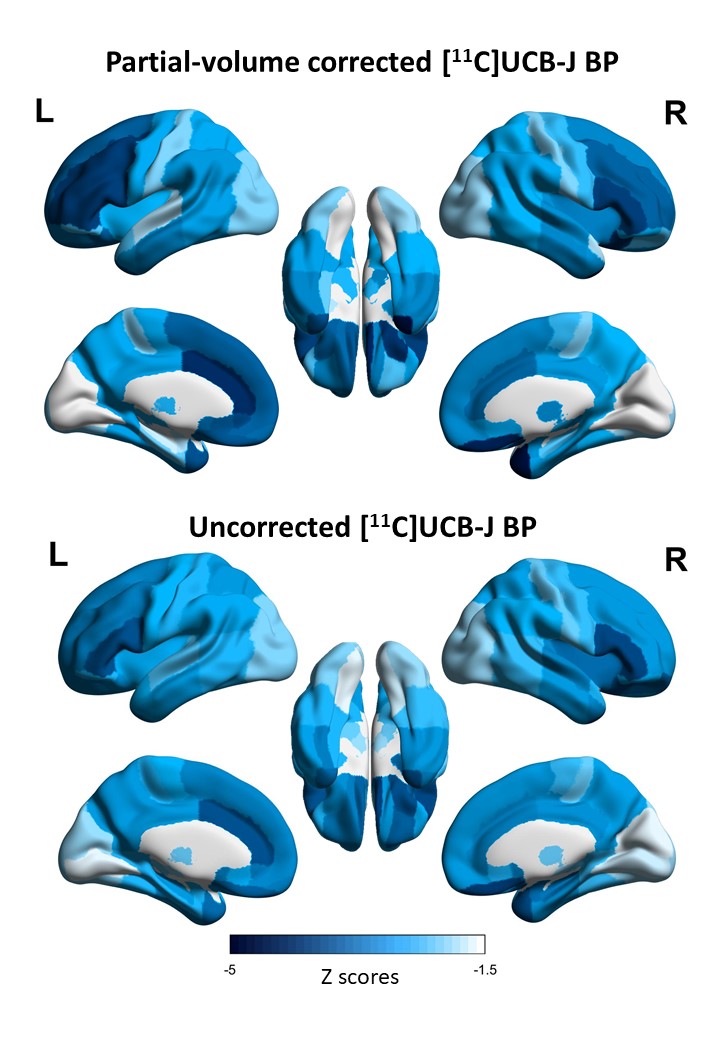


**Supplementary Figure 3. Whole brain correlation between regional z-scores for [^11^C]UCB-J binding potential (BP_ND_) without partial volume correction (y axis) and grey-matter volumes (GM, divided by total-intracranial volume; x axis).** Dots represent regional values for each subject, where individual dots for each region are represented with the same colour. The black line represents the association between the two modalities at the group level, while grey lines are correlations for individual patients.


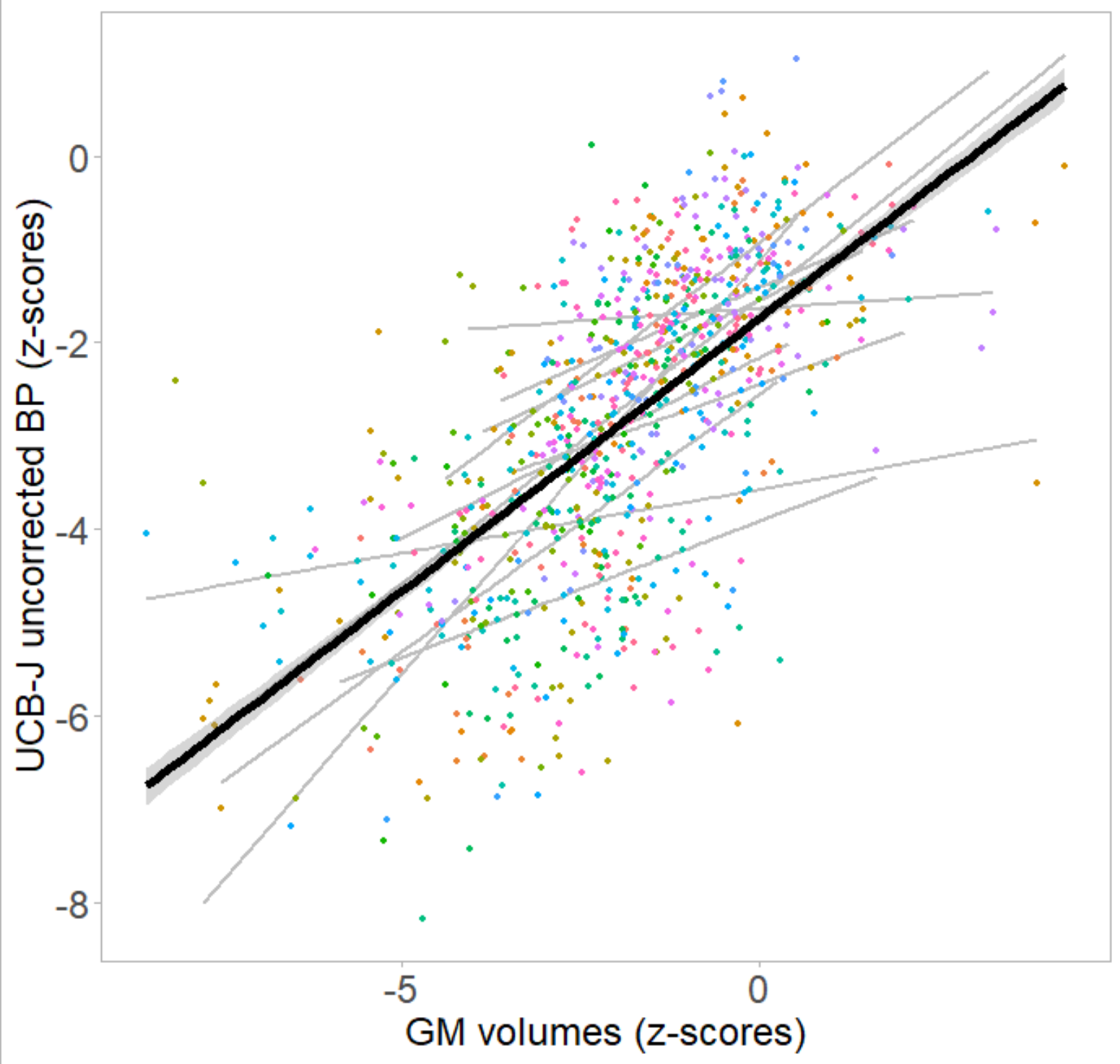


**Supplementary Table 1. Group comparisons of regional [^11^C]UCB-J binding potential (BP_ND_) with partial volume correction in Hammers atlas regions.** P < 0.05 are highlighted in yellow, while p < 0.001 are highlighted in red.

|  | **PV-corrected BP_ND_** | | | | **Statistical parameters** | | | |
| --- | --- | --- | --- | --- | --- | --- | --- | --- |
|  | **Mean CT** | **SD CT** | **Mean PT** | **SD PT** | **T value** | **Cohen's d** | **p** | **p FDR** |
| *Hippocampus R* | 1.84 | 0.28 | 1.17 | 0.39 | -5.12 | -1.96 | 0.0001 | 0.0005 |
| *Hippocampus L* | 1.84 | 0.27 | 1.36 | 0.53 | -2.84 | -1.14 | 0.0147 | 0.0157 |
| *Amygdala R* | 2.50 | 0.30 | 1.57 | 0.55 | -5.26 | -2.09 | 0.0002 | 0.0005 |
| *Amygdala L* | 2.46 | 0.32 | 1.86 | 0.49 | -3.80 | -1.48 | 0.0020 | 0.0029 |
| *Anterior temporal lobe medial part R* | 2.22 | 0.20 | 1.27 | 0.61 | -5.04 | -2.09 | 0.0004 | 0.0009 |
| *Anterior temporal lobe medial part L* | 2.19 | 0.21 | 1.35 | 0.59 | -4.59 | -1.89 | 0.0008 | 0.0013 |
| *Anterior temporal lobe lateral part R* | 2.50 | 0.23 | 1.79 | 0.62 | -3.68 | -1.52 | 0.0035 | 0.0047 |
| *Anterior temporal lobe lateral part L* | 2.51 | 0.29 | 1.65 | 0.62 | -4.40 | -1.78 | 0.0009 | 0.0014 |
| *Parahippocampal and ambient gyri R* | 1.72 | 0.25 | 0.98 | 0.40 | -5.64 | -2.20 | 0.0001 | 0.0004 |
| *Parahippocampal and ambient gyri L* | 1.52 | 0.24 | 0.97 | 0.59 | -2.94 | -1.20 | 0.0128 | 0.0140 |
| *Superior temporal gyrus posterior part R* | 2.84 | 0.20 | 2.21 | 0.43 | -4.62 | -1.86 | 0.0006 | 0.0011 |
| *Superior temporal gyrus posterior part L* | 2.76 | 0.29 | 2.19 | 0.30 | -5.23 | -1.90 | 0.0001 | 0.0004 |
| *Middle and inferior temporal gyrus R* | 2.66 | 0.24 | 1.89 | 0.44 | -5.50 | -2.19 | 0.0001 | 0.0005 |
| *Middle and inferior temporal gyrus L* | 2.66 | 0.29 | 1.74 | 0.58 | -5.02 | -2.02 | 0.0003 | 0.0007 |
| *Fusiform gyrus R* | 2.43 | 0.28 | 1.55 | 0.53 | -5.21 | -2.09 | 0.0002 | 0.0006 |
| *Fusiform gyrus L* | 2.38 | 0.25 | 1.56 | 0.55 | -4.76 | -1.93 | 0.0005 | 0.0010 |
| *Insula L* | 2.58 | 0.27 | 1.87 | 0.43 | -5.13 | -2.00 | 0.0002 | 0.0005 |
| *Insula R* | 2.69 | 0.26 | 1.79 | 0.39 | -6.91 | -2.68 | 0.0000 | 0.0002 |
| *Lateral remainder of occipital lobe L* | 2.74 | 0.27 | 2.16 | 0.41 | -4.25 | -1.66 | 0.0008 | 0.0013 |
| *Lateral remainder of occipital lobe R* | 2.71 | 0.24 | 2.22 | 0.40 | -3.71 | -1.46 | 0.0025 | 0.0035 |
| *Cingulate gyrus anterior part L* | 2.87 | 0.24 | 1.82 | 0.72 | -4.72 | -1.95 | 0.0006 | 0.0011 |
| *Cingulate gyrus anterior part R* | 2.82 | 0.27 | 1.85 | 0.55 | -5.52 | -2.23 | 0.0001 | 0.0005 |
| *Gyrus cinguli posterior part L* | 3.03 | 0.23 | 2.36 | 0.44 | -4.81 | -1.92 | 0.0004 | 0.0009 |
| *Gyrus cinguli posterior part R* | 3.01 | 0.24 | 2.27 | 0.44 | -5.21 | -2.07 | 0.0002 | 0.0006 |
| *Middle frontal gyrus L* | 2.89 | 0.24 | 1.86 | 0.63 | -5.26 | -2.16 | 0.0002 | 0.0007 |
| *Middle frontal gyrus R* | 2.93 | 0.24 | 2.06 | 0.46 | -5.93 | -2.38 | 0.0001 | 0.0004 |
| *Posterior temporal lobe L* | 2.69 | 0.26 | 1.94 | 0.34 | -6.49 | -2.47 | 0.0000 | 0.0002 |
| *Posterior temporal lobe R* | 2.70 | 0.23 | 2.08 | 0.34 | -5.47 | -2.12 | 0.0001 | 0.0004 |
| *Inferiolateral remainder of parietal lobe L* | 2.84 | 0.25 | 2.09 | 0.40 | -5.77 | -2.25 | 0.0001 | 0.0004 |
| *Inferiolateral remainder of parietal lobe R* | 2.80 | 0.23 | 2.17 | 0.37 | -5.27 | -2.07 | 0.0001 | 0.0005 |
| *Caudate nucleus L* | 2.99 | 0.34 | 2.55 | 0.44 | -2.94 | -1.12 | 0.0099 | 0.0114 |
| *Caudate nucleus R* | 2.97 | 0.34 | 2.51 | 0.44 | -2.97 | -1.17 | 0.0102 | 0.0116 |
| *Caudate nucleus L* | 3.84 | 0.33 | 3.03 | 0.77 | -3.31 | -1.35 | 0.0065 | 0.0079 |
| *Caudate nucleus R* | 3.97 | 0.38 | 2.93 | 0.88 | -3.61 | -1.54 | 0.0045 | 0.0060 |
| *Putamen L* | 3.93 | 0.38 | 3.34 | 0.41 | -4.12 | -1.51 | 0.0007 | 0.0011 |
| *Putamen R* | 3.86 | 0.35 | 3.20 | 0.44 | -4.38 | -1.66 | 0.0005 | 0.0010 |
| *Thalamus L* | 2.74 | 0.28 | 2.03 | 0.48 | -4.57 | -1.80 | 0.0005 | 0.0010 |
| *Thalamus R* | 2.86 | 0.31 | 1.86 | 0.79 | -4.07 | -1.67 | 0.0017 | 0.0026 |
| *Pallidum L* | 1.71 | 0.27 | 1.27 | 0.28 | -4.34 | -1.59 | 0.0004 | 0.0009 |
| *Pallidum R* | 1.96 | 0.25 | 1.57 | 0.29 | -3.93 | -1.46 | 0.0011 | 0.0017 |
| *Precentral gyrus L* | 2.67 | 0.19 | 2.14 | 0.35 | -4.72 | -1.88 | 0.0004 | 0.0009 |
| *Precentral gyrus R* | 2.67 | 0.19 | 2.20 | 0.29 | -4.97 | -1.92 | 0.0002 | 0.0006 |
| *Straight gyrus L* | 2.76 | 0.28 | 1.66 | 0.54 | -6.38 | -2.55 | 0.0000 | 0.0004 |
| *Straight gyrus R* | 2.71 | 0.25 | 1.61 | 0.62 | -5.75 | -2.35 | 0.0001 | 0.0005 |
| *Anterior orbital gyrus L* | 2.73 | 0.23 | 1.98 | 0.54 | -4.22 | -1.80 | 0.0017 | 0.0025 |
| *Anterior orbital gyrus R* | 2.78 | 0.23 | 2.21 | 0.89 | -2.07 | -0.87 | 0.0641 | 0.0649 |
| *Inferior frontal gyrus L* | 2.82 | 0.24 | 1.81 | 0.59 | -5.51 | -2.25 | 0.0002 | 0.0005 |
| *Inferior frontal gyrus R* | 2.83 | 0.21 | 1.95 | 0.58 | -4.92 | -2.03 | 0.0004 | 0.0009 |
| *Superior frontal gyrus L* | 2.84 | 0.25 | 1.93 | 0.49 | -5.85 | -2.35 | 0.0001 | 0.0004 |
| *Superior frontal gyrus R* | 2.87 | 0.26 | 2.04 | 0.42 | -6.00 | -2.36 | 0.0000 | 0.0004 |
| *Postcentral gyrus L* | 2.70 | 0.23 | 2.21 | 0.26 | -5.38 | -2.00 | 0.0001 | 0.0004 |
| *Postcentral gyrus R* | 2.68 | 0.22 | 2.21 | 0.25 | -5.26 | -1.96 | 0.0001 | 0.0004 |
| *Superior parietal gyrus L* | 3.12 | 0.25 | 2.40 | 0.33 | -6.49 | -2.46 | 0.0000 | 0.0002 |
| *Superior parietal gyrus R* | 3.08 | 0.23 | 2.45 | 0.29 | -6.24 | -2.36 | 0.0000 | 0.0002 |
| *Lingual gyrus L* | 2.84 | 0.35 | 2.49 | 0.38 | -2.55 | -0.94 | 0.0204 | 0.0213 |
| *Lingual gyrus R* | 2.96 | 0.30 | 2.55 | 0.38 | -3.15 | -1.19 | 0.0063 | 0.0079 |
| *Cuneus L* | 3.12 | 0.35 | 2.67 | 0.48 | -2.77 | -1.06 | 0.0144 | 0.0156 |
| *Cuneus R* | 3.14 | 0.33 | 2.69 | 0.37 | -3.47 | -1.29 | 0.0029 | 0.0040 |
| *Medial orbital gyrus L* | 2.63 | 0.28 | 1.62 | 0.62 | -5.16 | -2.09 | 0.0002 | 0.0007 |
| *Medial orbital gyrus R* | 2.66 | 0.30 | 1.70 | 0.67 | -4.61 | -1.87 | 0.0006 | 0.0011 |
| *Lateral orbital gyrus L* | 2.32 | 0.25 | 1.72 | 0.59 | -3.24 | -1.32 | 0.0073 | 0.0087 |
| *Lateral orbital gyrus R* | 2.43 | 0.25 | 1.72 | 0.70 | -3.26 | -1.34 | 0.0075 | 0.0088 |
| *Posterior orbital gyrus L* | 2.51 | 0.24 | 1.64 | 0.45 | -6.10 | -2.44 | 0.0000 | 0.0004 |
| *Posterior orbital gyrus R* | 2.53 | 0.23 | 1.55 | 0.61 | -5.18 | -2.13 | 0.0003 | 0.0007 |
| *Cingulate rostral L* | 2.73 | 0.31 | 1.57 | 1.13 | -3.35 | -1.40 | 0.0067 | 0.0081 |
| *Cingulate rostral R* | 2.57 | 0.38 | 1.52 | 1.01 | -3.36 | -1.38 | 0.0062 | 0.0078 |
| *Cingulate rostral L* | 3.11 | 0.57 | 1.08 | 1.80 | -3.49 | -1.52 | 0.0060 | 0.0077 |
| *Cingulate rostral R* | 2.92 | 0.59 | 1.26 | 2.09 | -2.48 | -1.08 | 0.0336 | 0.0345 |
| *Cingulate rostral L* | 3.41 | 0.38 | 1.91 | 1.72 | -2.87 | -1.21 | 0.0159 | 0.0168 |
| *Cingulate rostral R* | 3.26 | 0.42 | 2.02 | 1.35 | -2.98 | -1.24 | 0.0126 | 0.0140 |
| *Superior temporal gyrus anterior part L* | 2.25 | 0.26 | 1.65 | 0.37 | -4.70 | -1.88 | 0.0004 | 0.0009 |
| *Superior temporal gyrus anterior part R* | 2.34 | 0.26 | 1.84 | 0.39 | -3.86 | -1.50 | 0.0017 | 0.0025 |
| *Brainstem mid B* | 2.26 | 0.32 | 1.43 | 0.58 | -4.52 | -1.80 | 0.0006 | 0.0011 |
| *Brainstem pon B* | 0.96 | 0.15 | 0.75 | 0.17 | -3.56 | -1.32 | 0.0024 | 0.0034 |
| *Brainstem med B* | 0.67 | 0.15 | 0.45 | 0.20 | -3.20 | -1.22 | 0.0059 | 0.0077 |
| *Cerebellum gm R* | 2.13 | 0.21 | 1.72 | 0.15 | -6.63 | -2.24 | 0.0000 | 0.0000 |
| *Cerebellum gm L* | 1.44 | 0.73 | 1.71 | 0.19 | 1.73 | 0.51 | 0.0934 | 0.0934 |

Abbreviations: CT = controls, SD = standard deviation, PT = bvFTD patients, p = p value, FDR = false discovery rate correction, L = left, R = right.

**Supplementary Table 2. Group comparisons of regional [^11^C]UCB-J binding potential (BP_ND_) without partial volume correction in Hammers atlas regions.** P < 0.05 are highlighted in yellow, while p < 0.001 are highlighted in red.

|  | **Uncorrected BP_ND_** | | | | **Statistical parameters** | | | |  |
| --- | --- | --- | --- | --- | --- | --- | --- | --- | --- |
|  | **Mean CT** | **SD CT** | **Mean PT** | **SD PT** | **T value** | **Cohen's d** | **p** | **p FDR** | |
| *Hippocampus R* | 1.90 | 0.22 | 1.15 | 0.29 | -7.74 | -2.94 | 0.0000 | 0.0000 | |
| *Hippocampus L* | 1.89 | 0.21 | 1.27 | 0.36 | -5.39 | -2.13 | 0.0001 | 0.0002 | |
| *Amygdala R* | 2.34 | 0.24 | 1.48 | 0.45 | -6.02 | -2.40 | 0.0000 | 0.0001 | |
| *Amygdala L* | 2.32 | 0.25 | 1.67 | 0.42 | -4.74 | -1.87 | 0.0004 | 0.0004 | |
| *Anterior temporal lobe medial part R* | 1.92 | 0.19 | 1.18 | 0.40 | -5.88 | -2.38 | 0.0001 | 0.0001 | |
| *Anterior temporal lobe medial part L* | 1.90 | 0.20 | 1.20 | 0.43 | -5.09 | -2.06 | 0.0003 | 0.0003 | |
| *Anterior temporal lobe lateral part R* | 2.15 | 0.21 | 1.47 | 0.42 | -5.05 | -2.03 | 0.0003 | 0.0003 | |
| *Anterior temporal lobe lateral part L* | 2.20 | 0.27 | 1.43 | 0.46 | -5.15 | -2.03 | 0.0002 | 0.0003 | |
| *Parahippocampal and ambient gyri R* | 1.73 | 0.21 | 1.08 | 0.28 | -6.87 | -2.63 | 0.0000 | 0.0000 | |
| *Parahippocampal and ambient gyri L* | 1.59 | 0.20 | 1.07 | 0.33 | -4.78 | -1.88 | 0.0003 | 0.0004 | |
| *Superior temporal gyrus posterior part R* | 2.03 | 0.17 | 1.48 | 0.24 | -6.89 | -2.66 | 0.0000 | 0.0000 | |
| *Superior temporal gyrus posterior part L* | 1.97 | 0.22 | 1.45 | 0.24 | -5.96 | -2.20 | 0.0000 | 0.0001 | |
| *Middle and inferior temporal gyrus R* | 2.08 | 0.21 | 1.46 | 0.32 | -5.99 | -2.33 | 0.0000 | 0.0001 | |
| *Middle and inferior temporal gyrus L* | 2.06 | 0.24 | 1.36 | 0.40 | -5.32 | -2.09 | 0.0001 | 0.0002 | |
| *Fusiform gyrus R* | 2.14 | 0.24 | 1.40 | 0.43 | -5.37 | -2.13 | 0.0001 | 0.0002 | |
| *Fusiform gyrus L* | 2.11 | 0.21 | 1.41 | 0.42 | -5.26 | -2.12 | 0.0002 | 0.0003 | |
| *Insula L* | 1.88 | 0.18 | 1.36 | 0.32 | -5.05 | -2.00 | 0.0002 | 0.0003 | |
| *Insula R* | 2.01 | 0.19 | 1.35 | 0.29 | -7.05 | -2.75 | 0.0000 | 0.0000 | |
| *Lateral remainder of occipital lobe L* | 1.95 | 0.20 | 1.52 | 0.27 | -4.84 | -1.85 | 0.0002 | 0.0003 | |
| *Lateral remainder of occipital lobe R* | 1.94 | 0.18 | 1.57 | 0.26 | -4.22 | -1.63 | 0.0008 | 0.0009 | |
| *Cingulate gyrus anterior part L* | 2.16 | 0.21 | 1.33 | 0.47 | -5.68 | -2.30 | 0.0001 | 0.0002 | |
| *Cingulate gyrus anterior part R* | 2.05 | 0.23 | 1.33 | 0.38 | -5.90 | -2.32 | 0.0000 | 0.0001 | |
| *Gyrus cinguli posterior part L* | 2.32 | 0.21 | 1.67 | 0.36 | -5.54 | -2.18 | 0.0001 | 0.0002 | |
| *Gyrus cinguli posterior part R* | 2.26 | 0.21 | 1.61 | 0.30 | -6.56 | -2.53 | 0.0000 | 0.0001 | |
| *Middle frontal gyrus L* | 1.48 | 0.17 | 0.88 | 0.31 | -5.94 | -2.36 | 0.0001 | 0.0001 | |
| *Middle frontal gyrus R* | 1.58 | 0.18 | 1.05 | 0.28 | -5.78 | -2.26 | 0.0001 | 0.0001 | |
| *Posterior temporal lobe L* | 1.89 | 0.21 | 1.34 | 0.26 | -6.20 | -2.33 | 0.0000 | 0.0001 | |
| *Posterior temporal lobe R* | 1.92 | 0.19 | 1.46 | 0.24 | -5.63 | -2.14 | 0.0000 | 0.0001 | |
| *Inferiolateral remainder of parietal lobe L* | 1.86 | 0.20 | 1.29 | 0.29 | -5.96 | -2.29 | 0.0000 | 0.0001 | |
| *Inferiolateral remainder of parietal lobe R* | 1.89 | 0.18 | 1.42 | 0.25 | -5.64 | -2.17 | 0.0001 | 0.0001 | |
| *Caudate nucleus L* | 2.35 | 0.25 | 1.51 | 0.55 | -4.80 | -1.95 | 0.0005 | 0.0005 | |
| *Caudate nucleus R* | 2.30 | 0.26 | 1.44 | 0.49 | -5.46 | -2.18 | 0.0001 | 0.0002 | |
| *Caudate nucleus L* | 3.01 | 0.25 | 2.16 | 0.51 | -5.27 | -2.13 | 0.0002 | 0.0003 | |
| *Caudate nucleus R* | 3.03 | 0.25 | 2.12 | 0.52 | -5.51 | -2.22 | 0.0001 | 0.0002 | |
| *Putamen L* | 3.12 | 0.30 | 2.49 | 0.39 | -4.77 | -1.81 | 0.0002 | 0.0003 | |
| *Putamen R* | 3.19 | 0.29 | 2.55 | 0.40 | -4.83 | -1.85 | 0.0002 | 0.0003 | |
| *Thalamus L* | 2.12 | 0.19 | 1.69 | 0.28 | -4.70 | -1.81 | 0.0003 | 0.0004 | |
| *Thalamus R* | 2.11 | 0.19 | 1.67 | 0.24 | -5.34 | -2.01 | 0.0001 | 0.0001 | |
| *Pallidum L* | 1.33 | 0.20 | 1.04 | 0.19 | -4.13 | -1.48 | 0.0005 | 0.0006 | |
| *Pallidum R* | 1.54 | 0.19 | 1.27 | 0.19 | -3.98 | -1.43 | 0.0007 | 0.0008 | |
| *Precentral gyrus L* | 1.39 | 0.11 | 1.08 | 0.18 | -5.13 | -2.01 | 0.0002 | 0.0002 | |
| *Precentral gyrus R* | 1.43 | 0.12 | 1.15 | 0.16 | -5.21 | -2.00 | 0.0001 | 0.0002 | |
| *Straight gyrus L* | 2.44 | 0.26 | 1.45 | 0.39 | -7.57 | -2.94 | 0.0000 | 0.0000 | |
| *Straight gyrus R* | 2.38 | 0.23 | 1.44 | 0.39 | -7.53 | -2.97 | 0.0000 | 0.0000 | |
| *Anterior orbital gyrus L* | 2.00 | 0.18 | 1.40 | 0.32 | -5.91 | -2.34 | 0.0001 | 0.0001 | |
| *Anterior orbital gyrus R* | 2.07 | 0.16 | 1.46 | 0.26 | -7.13 | -2.79 | 0.0000 | 0.0000 | |
| *Inferior frontal gyrus L* | 2.09 | 0.20 | 1.32 | 0.34 | -7.09 | -2.80 | 0.0000 | 0.0000 | |
| *Inferior frontal gyrus R* | 2.15 | 0.17 | 1.45 | 0.30 | -7.14 | -2.82 | 0.0000 | 0.0000 | |
| *Superior frontal gyrus L* | 1.76 | 0.19 | 1.14 | 0.31 | -6.17 | -2.42 | 0.0000 | 0.0001 | |
| *Superior frontal gyrus R* | 1.76 | 0.19 | 1.19 | 0.26 | -6.65 | -2.54 | 0.0000 | 0.0000 | |
| *Postcentral gyrus L* | 1.54 | 0.14 | 1.18 | 0.21 | -5.20 | -2.01 | 0.0001 | 0.0002 | |
| *Postcentral gyrus R* | 1.54 | 0.14 | 1.22 | 0.17 | -5.47 | -2.05 | 0.0000 | 0.0001 | |
| *Superior parietal gyrus L* | 1.80 | 0.17 | 1.30 | 0.20 | -7.21 | -2.70 | 0.0000 | 0.0000 | |
| *Superior parietal gyrus R* | 1.81 | 0.16 | 1.34 | 0.19 | -7.27 | -2.69 | 0.0000 | 0.0000 | |
| *Lingual gyrus L* | 2.42 | 0.25 | 2.01 | 0.34 | -3.58 | -1.37 | 0.0027 | 0.0028 | |
| *Lingual gyrus R* | 2.43 | 0.23 | 2.03 | 0.32 | -3.73 | -1.43 | 0.0021 | 0.0022 | |
| *Cuneus L* | 2.51 | 0.25 | 2.00 | 0.38 | -4.04 | -1.57 | 0.0012 | 0.0013 | |
| *Cuneus R* | 2.50 | 0.27 | 2.04 | 0.38 | -3.63 | -1.40 | 0.0026 | 0.0027 | |
| *Medial orbital gyrus L* | 2.05 | 0.20 | 1.38 | 0.32 | -6.46 | -2.52 | 0.0000 | 0.0001 | |
| *Medial orbital gyrus R* | 2.05 | 0.21 | 1.40 | 0.31 | -6.27 | -2.42 | 0.0000 | 0.0001 | |
| *Lateral orbital gyrus L* | 1.91 | 0.20 | 1.39 | 0.28 | -5.57 | -2.14 | 0.0001 | 0.0001 | |
| *Lateral orbital gyrus R* | 1.98 | 0.19 | 1.45 | 0.34 | -4.90 | -1.94 | 0.0003 | 0.0004 | |
| *Posterior orbital gyrus L* | 2.02 | 0.20 | 1.40 | 0.26 | -6.99 | -2.66 | 0.0000 | 0.0000 | |
| *Posterior orbital gyrus R* | 2.07 | 0.18 | 1.36 | 0.33 | -6.73 | -2.68 | 0.0000 | 0.0001 | |
| *Cingulate rostral L* | 1.90 | 0.26 | 1.19 | 0.39 | -5.57 | -2.16 | 0.0001 | 0.0001 | |
| *Cingulate rostral R* | 1.65 | 0.24 | 1.03 | 0.37 | -5.12 | -1.99 | 0.0002 | 0.0002 | |
| *Cingulate rostral L* | 2.22 | 0.27 | 1.31 | 0.44 | -6.30 | -2.47 | 0.0000 | 0.0001 | |
| *Cingulate rostral R* | 2.24 | 0.28 | 1.29 | 0.48 | -6.11 | -2.41 | 0.0000 | 0.0001 | |
| *Cingulate rostral L* | 2.65 | 0.30 | 1.63 | 0.50 | -6.27 | -2.47 | 0.0000 | 0.0001 | |
| *Cingulate rostral R* | 2.43 | 0.27 | 1.53 | 0.53 | -5.36 | -2.15 | 0.0002 | 0.0002 | |
| *Superior temporal gyrus anterior part L* | 1.99 | 0.23 | 1.36 | 0.37 | -5.17 | -2.02 | 0.0002 | 0.0002 | |
| *Superior temporal gyrus anterior part R* | 2.06 | 0.23 | 1.48 | 0.33 | -5.23 | -2.02 | 0.0001 | 0.0002 | |
| *Brainstem mid B* | 1.07 | 0.12 | 0.84 | 0.22 | -3.27 | -1.31 | 0.0063 | 0.0064 | |
| *Brainstem pon B* | 0.91 | 0.14 | 0.73 | 0.15 | -3.51 | -1.29 | 0.0025 | 0.0026 | |
| *Brainstem med B* | 0.71 | 0.15 | 0.53 | 0.19 | -2.75 | -1.05 | 0.0146 | 0.0146 | |
| *Cerebellum gm R* | 2.02 | 0.20 | 1.59 | 0.17 | -6.55 | -2.30 | 0.0000 | 0.0000 | |
| *Cerebellum gm L* | 2.00 | 0.21 | 1.58 | 0.20 | -5.73 | -2.05 | 0.0000 | 0.0001 | |

Abbreviations: CT = controls, SD = standard deviation, PT = bvFTD patients, p = p value, FDR = false discovery rate correction, L = left, R = right.

**Supplementary Table 3. Spearman correlations in the bvFTD group between cognitive scores and [^11^C]UCB-J binding potential (BP_ND_) in aggregated cortical regions of interest.** Spearman rho (r) is reported alongside p value and Bayes factor (BF). * denotes p < 0.05 for frequentist tests of the null hypothesis. BF > 3 indicates strong evidence and BF > 10 indicates very strong evidence in favour of the alternate hypothesis (that there is a correlation) while BF < 1/3 denotes strong evidence for the null hypothesis (that there is no correlation). 1/3 < BF < 3 denotes insufficient evidence to support inferences for either hypothesis.

| **REGION** | **ACE-R** | **ATT/OR** | **MEM** | **FLUE** | **LANG** | **VISUO** | **IFS** | **IFS WM** | **MMSE** | **FTD-RS %** |
| --- | --- | --- | --- | --- | --- | --- | --- | --- | --- | --- |
| **Frontal L** | r=0.791 (0.002)* BF=56.2 | r=0.717 (0.007)* BF=21.3 | r=0.653 (0.015)* BF=7.9 | r=0.954 (< .001)* BF=4720.6 | r=0.795 (0.002)* BF=52.5 | r=0.816 (0.001)* BF=71.4 | r=0.754 (0.006)* BF=29.5 | r=0.74 (0.007)* BF=23.4 | r=0.736 (0.005)* BF=20.6 | r=-0.055 (0.446) BF=0.4 |
| **Frontal R** | r=0.118 (0.367) BF=0.4 | r=0.028 (0.467) BF=0.3 | r=0.027 (0.468) BF=0.4 | r=0.396 (0.114) BF=1.2 | r=0.354 (0.143) BF=1 | r=0.06 (0.431) BF=0.3 | r=0.17 (0.319) BF=0.7 | r=0.177 (0.312) BF=0.7 | r=0.133 (0.348) BF=0.4 | r=-0.382 (0.139) BF=0.2 |
| **Temporal L** | r=0.445 (0.086) BF=1.7 | r=0.422 (0.098) BF=1.5 | r=0.443 (0.086) BF=1.8 | r=0.691 (0.009)* BF=12.8 | r=0.612 (0.023)* BF=8.2 | r=0.581 (0.031)* BF=4.3 | r=0.547 (0.051) BF=3.4 | r=0.495 (0.073) BF=2.5 | r=0.382 (0.123) BF=1 | r=-0.042 (0.459) BF=0.4 |
| **Temporal R** | r=-0.245 (0.774) BF=0.2 | r=-0.244 (0.765) BF=0.2 | r=-0.174 (0.695) BF=0.2 | r=0.06 (0.431) BF=0.4 | r=0.032 (0.463) BF=0.4 | r=-0.088 (0.601) BF=0.2 | r=0.018 (0.48) BF=0.4 | r=-0.012 (0.513) BF=0.4 | r=-0.271 (0.79) BF=0.2 | r=-0.139 (0.354) BF=0.3 |
| **Cingulate L** | r=0.700 (0.008)* BF=10.7 | r=0.619 (0.021)* BF=4.9 | r=0.589 (0.028)* BF=3.6 | r=0.82 (< .001)* BF=46.5 | r=0.814 (0.001)* BF=88.9 | r=0.604 (0.025)* BF=6.1 | r=0.62 (0.028)* BF=2.3 | r=0.581 (0.039)* BF=1.9 | r=0.713 (0.007)* BF=9.1 | r=-0.067 (0.432) BF=0.5 |
| **Cingulate R** | r=0.073 (0.419) BF=0.5 | r=0.141 (0.34) BF=0.5 | r=-0.032 (0.537) BF=0.3 | r=0.3 (0.185) BF=0.9 | r=0.221 (0.257) BF=0.6 | r=0.041 (0.452) BF=0.3 | r=0.134 (0.356) BF=0.7 | r=0.116 (0.375) BF=0.8 | r=0.175 (0.304) BF=0.6 | r=-0.115 (0.379) BF=0.4 |
| **Insula L** | r=0.264 (0.217) BF=0.9 | r=0.314 (0.174) BF=0.9 | r=0.237 (0.241) BF=0.7 | r=0.562 (0.036)* BF=4.2 | r=0.446 (0.085) BF=2.4 | r=0.452 (0.082) BF=1.7 | r=0.48 (0.08) BF=3.8 | r=0.428 (0.109) BF=2.8 | r=0.257 (0.222) BF=0.7 | r=-0.273 (0.224) BF=0.2 |
| **Insula R** | r=-0.136 (0.663) BF=0.3 | r=-0.028 (0.533) BF=0.3 | r=-0.137 (0.656) BF=0.3 | r=0.138 (0.343) BF=0.5 | r=0.078 (0.41) BF=0.4 | r=-0.065 (0.575) BF=0.3 | r=0.061 (0.434) BF=0.6 | r=0.037 (0.46) BF=0.6 | r=-0.041 (0.548) BF=0.3 | r=-0.285 (0.214) BF=0.2 |
| **Parietal L** | r=0.591 (0.028)* BF=3.9 | r=0.351 (0.145) BF=1.3 | r=0.616 (0.022)* BF=5.7 | r=0.627 (0.02)* BF=5.9 | r=0.809 (0.001)* BF=30.5 | r=0.461 (0.077) BF=1.9 | r=0.632 (0.025)* BF=0.8 | r=0.575 (0.041)* BF=0.7 | r=0.377 (0.127) BF=1.2 | r=-0.042 (0.459) BF=1.8 |
| **Parietal R** | r=-0.109 (0.633) BF=0.3 | r=-0.216 (0.738) BF=0.3 | r=-0.027 (0.532) BF=0.4 | r=-0.157 (0.677) BF=0.3 | r=0.143 (0.338) BF=0.5 | r=-0.23 (0.752) BF=0.2 | r=-0.14 (0.65) BF=0.2 | r=-0.22 (0.729) BF=0.2 | r=-0.216 (0.738) BF=0.3 | r=0.079 (0.594) BF=3 |
| **Occipital L** | r=-0.255 (0.783) BF=0.2 | r=-0.145 (0.665) BF=0.2 | r=-0.251 (0.772) BF=0.2 | r=-0.051 (0.559) BF=0.3 | r=-0.299 (0.814) BF=0.2 | r=0.000  (0.5) BF=0.3 | r=-0.201 (0.711) BF=0.3 | r=-0.196 (0.706) BF=0.3 | r=-0.28 (0.798) BF=0.2 | r=0.091 (0.607) BF=0.5 |
| **Occipital R** | r=-0.355 (0.863) BF=0.2 | r=-0.248 (0.769) BF=0.2 | r=-0.315 (0.827) BF=0.2 | r=-0.147 (0.667) BF=0.2 | r=-0.34 (0.847) BF=0.2 | r=-0.138 (0.657) BF=0.3 | r=-0.292 (0.793) BF=0.2 | r=-0.287 (0.79) BF=0.3 | r=-0.372 (0.87) BF=0.2 | r=0.236 (0.754) BF=0.6 |

Abbreviations: ACE-R = Addenbrooke's cognitive examination revised; ATT/OR= attention/orientation score; MEM = memory; FLUE = fluency; LANG = language; VISUO= visuospatial; IFS = INECO frontal screening; MMSE = mini-mental state examination; WM = working memory; FTD-RS = frontotemporal dementia rating scale; L = left; R = right.

**Supplementary Table 4. Comparisons of group-average regional grey-matter volume (GM) z-scores with regional [^11^C]UCB-J binding potential z-scores with (PVC) and without (Uncorr) partial volume correction.** Regions with lower mean [^11^C]UCB-J z-scores than grey-matter volume z-scores are highlighted in yellow.

|  | **Mean Zscore GM** | **SD Zscore GM** | **Mean Zscore UCB-J PVC** | **SD Zscore UCB-J PVC** | **UCB-J PVC < GM** | **Mean Zscore UCB-J Uncorr** | **SD Zscore UCB-J Uncorr** | **UCB-J Uncorr < GM** |
| --- | --- | --- | --- | --- | --- | --- | --- | --- |
| *Hippocampus R* | -1.85 | 2.40 | -2.39 | 1.40 | 1 | -3.43 | 1.31 | 1 |
| *Hippocampus L* | -1.94 | 2.87 | -1.80 | 2.00 | 0 | -3.00 | 1.72 | 1 |
| *Amygdala R* | -2.74 | 2.15 | -3.09 | 1.83 | 1 | -3.57 | 1.85 | 1 |
| *Amygdala L* | -2.09 | 2.25 | -1.91 | 1.53 | 0 | -2.63 | 1.71 | 1 |
| *Anterior temporal lobe medial part R* | -2.40 | 1.71 | -4.68 | 3.01 | 1 | -3.92 | 2.11 | 1 |
| *Anterior temporal lobe medial part L* | -1.79 | 1.66 | -3.97 | 2.79 | 1 | -3.54 | 2.21 | 1 |
| *Anterior temporal lobe lateral part R* | -2.25 | 2.03 | -3.11 | 2.72 | 1 | -3.22 | 2.01 | 1 |
| *Anterior temporal lobe lateral part L* | -1.94 | 1.66 | -2.97 | 2.14 | 1 | -2.83 | 1.70 | 1 |
| *Parahippocampal and ambient gyri R* | -2.57 | 2.04 | -2.90 | 1.57 | 1 | -3.14 | 1.37 | 1 |
| *Parahippocampal and ambient gyri L* | -2.16 | 1.69 | -2.24 | 2.44 | 1 | -2.57 | 1.66 | 1 |
| *Superior temporal gyrus posterior part R* | -1.81 | 1.66 | -3.09 | 2.12 | 1 | -3.30 | 1.44 | 1 |
| *Superior temporal gyrus posterior part L* | -1.66 | 2.59 | -1.94 | 1.04 | 1 | -2.33 | 1.11 | 1 |
| *Middle and inferior temporal gyrus R* | -2.82 | 2.19 | -3.21 | 1.82 | 1 | -3.00 | 1.52 | 1 |
| *Middle and inferior temporal gyrus L* | -3.13 | 2.52 | -3.22 | 2.02 | 1 | -2.87 | 1.66 | 0 |
| *Fusiform gyrus R* | -1.32 | 1.89 | -3.19 | 1.92 | 1 | -3.09 | 1.79 | 1 |
| *Fusiform gyrus L* | -1.60 | 1.70 | -3.24 | 2.16 | 1 | -3.39 | 2.03 | 1 |
| *Insula L* | -1.80 | 1.31 | -2.66 | 1.59 | 1 | -2.85 | 1.75 | 1 |
| *Insula R* | -2.35 | 1.65 | -3.40 | 1.49 | 1 | -3.58 | 1.55 | 1 |
| *Lateral remainder of occipital lobe L* | -1.70 | 1.12 | -2.16 | 1.54 | 1 | -2.18 | 1.34 | 1 |
| *Lateral remainder of occipital lobe R* | -1.68 | 1.24 | -2.03 | 1.68 | 1 | -2.05 | 1.47 | 1 |
| *Cingulate gyrus anterior part L* | -2.21 | 1.43 | -4.34 | 2.97 | 1 | -3.92 | 2.19 | 1 |
| *Cingulate gyrus anterior part R* | -1.16 | 0.75 | -3.63 | 2.08 | 1 | -3.21 | 1.68 | 1 |
| *Gyrus cinguli posterior part L* | -0.89 | 1.58 | -2.94 | 1.92 | 1 | -3.03 | 1.69 | 1 |
| *Gyrus cinguli posterior part R* | -1.39 | 1.54 | -3.07 | 1.84 | 1 | -3.13 | 1.43 | 1 |
| *Middle frontal gyrus L* | -2.34 | 1.13 | -4.19 | 2.56 | 1 | -3.41 | 1.79 | 1 |
| *Middle frontal gyrus R* | -2.03 | 0.93 | -3.71 | 1.96 | 1 | -3.03 | 1.61 | 1 |
| *Posterior temporal lobe L* | -2.19 | 1.50 | -2.91 | 1.33 | 1 | -2.59 | 1.22 | 1 |
| *Posterior temporal lobe R* | -2.30 | 1.32 | -2.70 | 1.49 | 1 | -2.47 | 1.29 | 1 |
| *Inferiolateral remainder of parietal lobe L* | -2.25 | 1.84 | -2.97 | 1.58 | 1 | -2.79 | 1.40 | 1 |
| *Inferiolateral remainder of parietal lobe R* | -1.35 | 1.13 | -2.80 | 1.63 | 1 | -2.65 | 1.41 | 1 |
| *Caudate nucleus L* | -4.23 | 2.67 | -1.30 | 1.31 | 0 | -3.36 | 2.22 | 0 |
| *Caudate nucleus R* | -4.42 | 2.42 | -1.33 | 1.26 | 0 | -3.32 | 1.91 | 0 |
| *Caudate nucleus L* | -1.76 | 1.96 | -2.47 | 2.38 | 1 | -3.46 | 2.07 | 1 |
| *Caudate nucleus R* | -1.88 | 1.57 | -2.77 | 2.34 | 1 | -3.61 | 2.07 | 1 |
| *Putamen L* | -1.86 | 1.33 | -1.57 | 1.08 | 0 | -2.10 | 1.30 | 1 |
| *Putamen R* | -1.97 | 1.46 | -1.90 | 1.27 | 0 | -2.25 | 1.40 | 1 |
| *Thalamus L* | -1.83 | 0.90 | -2.53 | 1.71 | 1 | -2.26 | 1.45 | 1 |
| *Thalamus R* | -1.73 | 0.98 | -3.25 | 2.57 | 1 | -2.28 | 1.25 | 1 |
| *Pallidum L* | 0.30 | 1.20 | -1.63 | 1.05 | 1 | -1.45 | 0.96 | 1 |
| *Pallidum R* | 0.44 | 1.59 | -1.58 | 1.15 | 1 | -1.41 | 0.97 | 1 |
| *Precentral gyrus L* | -2.64 | 1.70 | -2.79 | 1.85 | 1 | -2.65 | 1.58 | 1 |
| *Precentral gyrus R* | -2.07 | 1.21 | -2.42 | 1.47 | 1 | -2.41 | 1.39 | 1 |
| *Straight gyrus L* | -2.18 | 0.92 | -3.87 | 1.90 | 1 | -3.76 | 1.51 | 1 |
| *Straight gyrus R* | -2.07 | 1.37 | -4.45 | 2.48 | 1 | -4.14 | 1.70 | 1 |
| *Anterior orbital gyrus L* | -3.53 | 2.30 | -3.29 | 2.38 | 0 | -3.32 | 1.74 | 0 |
| *Anterior orbital gyrus R* | -3.66 | 2.00 | -2.41 | 3.80 | 0 | -3.72 | 1.60 | 1 |
| *Inferior frontal gyrus L* | -2.10 | 1.12 | -4.15 | 2.41 | 1 | -3.93 | 1.72 | 1 |
| *Inferior frontal gyrus R* | -2.10 | 1.25 | -4.17 | 2.73 | 1 | -3.99 | 1.73 | 1 |
| *Superior frontal gyrus L* | -2.52 | 1.25 | -3.67 | 1.97 | 1 | -3.29 | 1.64 | 1 |
| *Superior frontal gyrus R* | -2.63 | 1.35 | -3.21 | 1.65 | 1 | -3.05 | 1.37 | 1 |
| *Postcentral gyrus L* | -1.80 | 1.34 | -2.16 | 1.16 | 1 | -2.55 | 1.49 | 1 |
| *Postcentral gyrus R* | -1.76 | 1.39 | -2.13 | 1.16 | 1 | -2.26 | 1.20 | 1 |
| *Superior parietal gyrus L* | -2.24 | 1.65 | -2.82 | 1.28 | 1 | -2.98 | 1.20 | 1 |
| *Superior parietal gyrus R* | -2.10 | 1.86 | -2.71 | 1.28 | 1 | -2.88 | 1.14 | 1 |
| *Lingual gyrus L* | -1.21 | 1.12 | -0.98 | 1.09 | 0 | -1.63 | 1.36 | 1 |
| *Lingual gyrus R* | -1.31 | 0.80 | -1.36 | 1.27 | 1 | -1.75 | 1.41 | 1 |
| *Cuneus L* | -1.09 | 1.19 | -1.27 | 1.37 | 1 | -2.00 | 1.50 | 1 |
| *Cuneus R* | -0.74 | 1.48 | -1.37 | 1.13 | 1 | -1.72 | 1.43 | 1 |
| *Medial orbital gyrus L* | -2.89 | 1.38 | -3.55 | 2.18 | 1 | -3.31 | 1.57 | 1 |
| *Medial orbital gyrus R* | -2.90 | 1.51 | -3.25 | 2.25 | 1 | -3.02 | 1.45 | 1 |
| *Lateral orbital gyrus L* | -2.55 | 1.79 | -2.35 | 2.31 | 0 | -2.60 | 1.40 | 1 |
| *Lateral orbital gyrus R* | -1.31 | 1.17 | -2.85 | 2.82 | 1 | -2.78 | 1.76 | 1 |
| *Posterior orbital gyrus L* | -2.90 | 1.16 | -3.66 | 1.87 | 1 | -3.10 | 1.31 | 1 |
| *Posterior orbital gyrus R* | -3.01 | 1.82 | -4.30 | 2.67 | 1 | -3.96 | 1.83 | 1 |
| *Cingulate rostral L* | -1.48 | 1.32 | -3.76 | 3.66 | 1 | -2.76 | 1.51 | 1 |
| *Cingulate rostral R* | -1.34 | 1.85 | -2.77 | 2.66 | 1 | -2.58 | 1.53 | 1 |
| *Cingulate rostral L* | -1.73 | 1.39 | -3.56 | 3.16 | 1 | -3.37 | 1.65 | 1 |
| *Cingulate rostral R* | -1.73 | 1.36 | -2.83 | 3.55 | 1 | -3.32 | 1.68 | 1 |
| *Cingulate rostral L* | -1.32 | 1.05 | -4.00 | 4.56 | 1 | -3.42 | 1.68 | 1 |
| *Cingulate rostral R* | -0.64 | 1.55 | -2.97 | 3.24 | 1 | -3.29 | 1.93 | 1 |
| *Superior temporal gyrus anterior part L* | -2.69 | 2.03 | -2.33 | 1.43 | 0 | -2.71 | 1.61 | 1 |
| *Superior temporal gyrus anterior part R* | -1.47 | 1.41 | -1.89 | 1.48 | 1 | -2.52 | 1.46 | 1 |
| *Brainstem mid B* | -0.43 | 0.93 | -2.62 | 1.80 | 1 | -1.96 | 1.88 | 1 |
| *Brainstem pon B* | 0.45 | 1.94 | -1.40 | 1.12 | 1 | -1.33 | 1.07 | 1 |
| *Brainstem med B* | 0.09 | 1.57 | -1.45 | 1.35 | 1 | -1.22 | 1.32 | 1 |
| *Cerebellum gm R* | -1.43 | 2.02 | -1.95 | 0.71 | 1 | -2.15 | 0.87 | 1 |
| *Cerebellum gm L* | -1.42 | 1.77 | 0.37 | 0.25 | 0 | -1.99 | 0.94 | 1 |

Abbreviations: PVC=partial-volume correction, SD = standard deviation, Uncorr =binding potential without partial volume correction; GM=grey-matter volume; L = left, R = right.

**Supplementary Table 5. Regional results of logistic regression models with [^11^C]UCB-J binding potential (BP_ND_) from data without partial-volume correction and grey-matter volume as predictors of groups (controls vs. patients).** The last five columns describe comparisons between each regional model with grey-matter volume only and the same model also including [^11^C]UCB-J BP_ND_ as a predictor. P < 0.05 are highlighted in yellow, while p < 0.001 are highlighted in red.

|  | **UCB-J BP_ND_ term** | | | **Grey-matter volume term** | | | **Model comparison** | | | | |
| --- | --- | --- | --- | --- | --- | --- | --- | --- | --- | --- | --- |
|  | **Estimate** | **Std Err** | **p** | **Estimate** | **Std Err** | **p** | **F ANOVA** | **p FDR** | **AIC MRI** | **AIC MRI + UCB-J** | **Delta AIC** |
| *Hippocampus R* | -0.9025 | 0.1322 | 0.0000 | -0.0001 | 0.0003 | 0.8444 | 46.62 | 0.0000 | 43.11 | 13.95 | 29.17 |
| *Hippocampus L* | -0.9147 | 0.1785 | 0.0000 | 0.0000 | 0.0005 | 0.9288 | 26.27 | 0.0001 | 44.50 | 25.96 | 18.54 |
| *Amygdala R* | -0.6173 | 0.1500 | 0.0002 | -0.0008 | 0.0008 | 0.3379 | 16.93 | 0.0006 | 31.64 | 19.27 | 12.37 |
| *Amygdala L* | -0.6563 | 0.1789 | 0.0009 | -0.0006 | 0.0007 | 0.3821 | 13.45 | 0.0014 | 39.82 | 30.06 | 9.77 |
| *Anterior temporal lobe medial part R* | -0.6829 | 0.1546 | 0.0001 | -0.0002 | 0.0001 | 0.1343 | 19.51 | 0.0003 | 31.38 | 17.20 | 14.18 |
| *Anterior temporal lobe medial part L* | -0.7699 | 0.1770 | 0.0001 | -0.0001 | 0.0001 | 0.7003 | 18.91 | 0.0003 | 39.07 | 25.31 | 13.77 |
| *Anterior temporal lobe lateral part R* | -0.6917 | 0.1809 | 0.0006 | -0.0002 | 0.0002 | 0.3445 | 14.63 | 0.0010 | 36.42 | 25.75 | 10.67 |
| *Anterior temporal lobe lateral part L* | -0.7091 | 0.1993 | 0.0012 | 0.0000 | 0.0003 | 0.9810 | 12.66 | 0.0017 | 37.07 | 27.93 | 9.15 |
| *Parahippocampal and ambient gyri R* | -0.8885 | 0.2024 | 0.0001 | -0.0002 | 0.0003 | 0.5468 | 19.28 | 0.0003 | 32.60 | 18.58 | 14.02 |
| *Parahippocampal and ambient gyri L* | -0.7053 | 0.2403 | 0.0060 | -0.0005 | 0.0003 | 0.1428 | 8.61 | 0.0070 | 34.45 | 28.64 | 5.81 |
| *Superior temporal gyrus posterior part R* | -1.1485 | 0.2080 | 0.0000 | 0.0000 | 0.0001 | 0.8643 | 30.48 | 0.0000 | 38.87 | 17.86 | 21.01 |
| *Superior temporal gyrus posterior part L* | -1.1258 | 0.2254 | 0.0000 | 0.0001 | 0.0001 | 0.5614 | 24.94 | 0.0001 | 45.65 | 27.93 | 17.72 |
| *Middle and inferior temporal gyrus R* | -0.7666 | 0.2152 | 0.0011 | -0.0001 | 0.0001 | 0.2755 | 12.70 | 0.0017 | 31.11 | 21.93 | 9.18 |
| *Middle and inferior temporal gyrus L* | -0.5795 | 0.1945 | 0.0054 | -0.0001 | 0.0001 | 0.1764 | 8.88 | 0.0065 | 30.95 | 24.91 | 6.04 |
| *Fusiform gyrus R* | -0.8176 | 0.1501 | 0.0000 | 0.0002 | 0.0003 | 0.5786 | 29.69 | 0.0000 | 45.88 | 25.32 | 20.56 |
| *Fusiform gyrus L* | -0.7569 | 0.1547 | 0.0000 | -0.0001 | 0.0002 | 0.5723 | 23.95 | 0.0001 | 41.73 | 24.63 | 17.10 |
| *Insula L* | -0.7969 | 0.2032 | 0.0004 | -0.0002 | 0.0001 | 0.0808 | 15.37 | 0.0008 | 36.17 | 24.95 | 11.23 |
| *Insula R* | -0.8398 | 0.1652 | 0.0000 | -0.0001 | 0.0001 | 0.1428 | 25.84 | 0.0001 | 31.52 | 13.25 | 18.28 |
| *Lateral remainder of occipital lobe L* | -0.7688 | 0.2668 | 0.0069 | -0.0001 | 0.0000 | 0.1151 | 8.30 | 0.0077 | 35.99 | 30.45 | 5.54 |
| *Lateral remainder of occipital lobe R* | -0.7888 | 0.3111 | 0.0161 | -0.0001 | 0.0000 | 0.1084 | 6.43 | 0.0168 | 37.42 | 33.55 | 3.87 |
| *Cingulate gyrus anterior part L* | -0.6233 | 0.1587 | 0.0004 | -0.0002 | 0.0002 | 0.3405 | 15.43 | 0.0008 | 31.13 | 19.86 | 11.27 |
| *Cingulate gyrus anterior part R* | -0.8039 | 0.1499 | 0.0000 | 0.0000 | 0.0001 | 0.7680 | 28.74 | 0.0000 | 42.45 | 22.44 | 20.01 |
| *Gyrus cinguli posterior part L* | -0.9701 | 0.1580 | 0.0000 | 0.0001 | 0.0001 | 0.3364 | 37.68 | 0.0000 | 48.95 | 24.06 | 24.88 |
| *Gyrus cinguli posterior part R* | -0.9354 | 0.1589 | 0.0000 | 0.0000 | 0.0002 | 0.8396 | 34.64 | 0.0000 | 43.48 | 20.19 | 23.29 |
| *Middle frontal gyrus L* | -0.7083 | 0.1954 | 0.0010 | -0.0001 | 0.0000 | 0.0316 | 13.14 | 0.0015 | 25.78 | 16.25 | 9.53 |
| *Middle frontal gyrus R* | -0.7718 | 0.2116 | 0.0009 | -0.0001 | 0.0000 | 0.0363 | 13.31 | 0.0015 | 28.82 | 19.16 | 9.66 |
| *Posterior temporal lobe L* | -0.7918 | 0.2212 | 0.0011 | -0.0001 | 0.0000 | 0.1395 | 12.81 | 0.0017 | 32.20 | 22.93 | 9.27 |
| *Posterior temporal lobe R* | -0.7321 | 0.2769 | 0.0124 | -0.0001 | 0.0000 | 0.0565 | 6.99 | 0.0133 | 28.53 | 24.16 | 4.38 |
| *Inferiolateral remainder of parietal lobe L* | -0.8596 | 0.2227 | 0.0005 | 0.0000 | 0.0000 | 0.3735 | 14.90 | 0.0010 | 34.72 | 23.85 | 10.87 |
| *Inferiolateral remainder of parietal lobe R* | -1.0676 | 0.2338 | 0.0001 | 0.0000 | 0.0000 | 0.5192 | 20.84 | 0.0002 | 41.54 | 26.45 | 15.08 |
| *Caudate nucleus L* | -0.2730 | 0.1706 | 0.1191 | -0.0005 | 0.0002 | 0.0129 | 2.56 | 0.1207 | 21.04 | 20.89 | 0.15 |
| *Caudate nucleus R* | -0.2477 | 0.1770 | 0.1710 | -0.0006 | 0.0002 | 0.0078 | 1.96 | 0.1710 | 15.87 | 16.33 | 0.00 |
| *Caudate nucleus L* | -0.7058 | 0.1447 | 0.0000 | 0.0009 | 0.0036 | 0.7994 | 23.80 | 0.0001 | 41.58 | 24.57 | 17.01 |
| *Caudate nucleus R* | -0.5858 | 0.1277 | 0.0001 | -0.0032 | 0.0038 | 0.3956 | 21.05 | 0.0002 | 37.19 | 21.97 | 15.23 |
| *Putamen L* | -0.4954 | 0.1712 | 0.0067 | -0.0004 | 0.0002 | 0.0520 | 8.38 | 0.0076 | 35.43 | 29.83 | 5.60 |
| *Putamen R* | -0.5152 | 0.1772 | 0.0065 | -0.0005 | 0.0003 | 0.0821 | 8.45 | 0.0074 | 35.04 | 29.37 | 5.67 |
| *Thalamus L* | -0.7137 | 0.2047 | 0.0014 | -0.0005 | 0.0001 | 0.0026 | 12.16 | 0.0019 | 31.89 | 23.14 | 8.75 |
| *Thalamus R* | -0.8264 | 0.2096 | 0.0004 | -0.0005 | 0.0002 | 0.0084 | 15.54 | 0.0008 | 34.33 | 22.98 | 11.35 |
| *Pallidum L* | -1.1321 | 0.2871 | 0.0004 | 0.0009 | 0.0016 | 0.6066 | 15.55 | 0.0008 | 52.48 | 41.12 | 11.36 |
| *Pallidum R* | -1.1224 | 0.2940 | 0.0006 | 0.0016 | 0.0019 | 0.4013 | 14.57 | 0.0010 | 52.03 | 41.40 | 10.62 |
| *Precentral gyrus L* | -1.0408 | 0.4167 | 0.0177 | -0.0001 | 0.0001 | 0.0410 | 6.24 | 0.0181 | 28.07 | 24.38 | 3.69 |
| *Precentral gyrus R* | -1.1861 | 0.3991 | 0.0055 | -0.0001 | 0.0000 | 0.0304 | 8.83 | 0.0065 | 31.00 | 25.00 | 6.00 |
| *Straight gyrus L* | -0.5607 | 0.1155 | 0.0000 | -0.0005 | 0.0003 | 0.0885 | 23.56 | 0.0001 | 26.21 | 9.36 | 16.86 |
| *Straight gyrus R* | -0.6672 | 0.1138 | 0.0000 | -0.0003 | 0.0002 | 0.2767 | 34.40 | 0.0000 | 32.64 | 9.47 | 23.17 |
| *Anterior orbital gyrus L* | -0.6574 | 0.1782 | 0.0008 | -0.0005 | 0.0002 | 0.0086 | 13.62 | 0.0014 | 24.15 | 14.26 | 9.90 |
| *Anterior orbital gyrus R* | -0.7436 | 0.1875 | 0.0004 | -0.0005 | 0.0002 | 0.0105 | 15.73 | 0.0008 | 18.76 | 7.27 | 11.49 |
| *Inferior frontal gyrus L* | -0.7564 | 0.1531 | 0.0000 | -0.0001 | 0.0001 | 0.2591 | 24.39 | 0.0001 | 29.60 | 12.22 | 17.38 |
| *Inferior frontal gyrus R* | -0.8829 | 0.1720 | 0.0000 | -0.0001 | 0.0001 | 0.3973 | 26.35 | 0.0001 | 30.95 | 12.37 | 18.59 |
| *Superior frontal gyrus L* | -0.6789 | 0.2086 | 0.0026 | -0.0001 | 0.0000 | 0.0524 | 10.59 | 0.0033 | 24.24 | 16.76 | 7.48 |
| *Superior frontal gyrus R* | -0.7303 | 0.2259 | 0.0028 | -0.0001 | 0.0000 | 0.0606 | 10.45 | 0.0034 | 23.86 | 16.49 | 7.37 |
| *Postcentral gyrus L* | -1.2557 | 0.3868 | 0.0027 | -0.0001 | 0.0001 | 0.5604 | 10.54 | 0.0033 | 36.39 | 28.95 | 7.44 |
| *Postcentral gyrus R* | -1.3080 | 0.3968 | 0.0023 | -0.0001 | 0.0001 | 0.4126 | 10.87 | 0.0030 | 37.38 | 29.68 | 7.71 |
| *Superior parietal gyrus L* | -1.0415 | 0.2164 | 0.0000 | -0.0001 | 0.0000 | 0.1247 | 23.16 | 0.0001 | 32.98 | 16.38 | 16.60 |
| *Superior parietal gyrus R* | -1.2072 | 0.2406 | 0.0000 | 0.0000 | 0.0000 | 0.4077 | 25.17 | 0.0001 | 36.72 | 18.86 | 17.86 |
| *Lingual gyrus L* | -0.6114 | 0.2310 | 0.0123 | -0.0002 | 0.0001 | 0.1386 | 7.01 | 0.0133 | 43.54 | 39.15 | 4.39 |
| *Lingual gyrus R* | -0.6111 | 0.2334 | 0.0132 | -0.0003 | 0.0002 | 0.0510 | 6.85 | 0.0140 | 40.25 | 35.99 | 4.25 |
| *Cuneus L* | -0.6925 | 0.1965 | 0.0013 | -0.0001 | 0.0001 | 0.3433 | 12.42 | 0.0018 | 45.41 | 36.45 | 8.96 |
| *Cuneus R* | -0.7118 | 0.1994 | 0.0011 | 0.0000 | 0.0001 | 0.8382 | 12.75 | 0.0017 | 49.94 | 40.72 | 9.22 |
| *Medial orbital gyrus L* | -0.5888 | 0.1711 | 0.0016 | -0.0007 | 0.0002 | 0.0090 | 11.84 | 0.0021 | 20.46 | 11.97 | 8.49 |
| *Medial orbital gyrus R* | -0.5806 | 0.1603 | 0.0010 | -0.0007 | 0.0002 | 0.0033 | 13.12 | 0.0015 | 21.98 | 12.46 | 9.51 |
| *Lateral orbital gyrus L* | -0.7086 | 0.2017 | 0.0013 | -0.0010 | 0.0004 | 0.0189 | 12.34 | 0.0018 | 30.32 | 21.43 | 8.90 |
| *Lateral orbital gyrus R* | -0.8566 | 0.1949 | 0.0001 | -0.0003 | 0.0003 | 0.3198 | 19.31 | 0.0003 | 42.31 | 28.26 | 14.04 |
| *Posterior orbital gyrus L* | -0.6081 | 0.1658 | 0.0009 | -0.0008 | 0.0002 | 0.0013 | 13.46 | 0.0014 | 17.15 | 7.38 | 9.77 |
| *Posterior orbital gyrus R* | -0.7086 | 0.1690 | 0.0002 | -0.0004 | 0.0002 | 0.0747 | 17.59 | 0.0005 | 24.53 | 11.69 | 12.84 |
| *Cingulate rostral L* | -0.8976 | 0.1964 | 0.0001 | 0.0007 | 0.0010 | 0.4633 | 20.88 | 0.0002 | 40.97 | 25.86 | 15.11 |
| *Cingulate rostral R* | -0.8321 | 0.1763 | 0.0000 | 0.0001 | 0.0009 | 0.9399 | 22.28 | 0.0001 | 45.48 | 29.45 | 16.03 |
| *Cingulate rostral L* | -0.6260 | 0.1211 | 0.0000 | -0.0047 | 0.0053 | 0.3820 | 26.73 | 0.0001 | 37.88 | 19.06 | 18.82 |
| *Cingulate rostral R* | -0.5497 | 0.1022 | 0.0000 | -0.0096 | 0.0052 | 0.0733 | 28.93 | 0.0000 | 37.57 | 17.45 | 20.12 |
| *Cingulate rostral L* | -0.6305 | 0.1118 | 0.0000 | 0.0001 | 0.0009 | 0.8723 | 31.81 | 0.0000 | 41.51 | 19.75 | 21.76 |
| *Cingulate rostral R* | -0.7376 | 0.1084 | 0.0000 | 0.0017 | 0.0010 | 0.0995 | 46.31 | 0.0000 | 50.83 | 21.80 | 29.03 |
| *Superior temporal gyrus anterior part L* | -0.6016 | 0.2274 | 0.0124 | -0.0003 | 0.0002 | 0.1990 | 7.00 | 0.0133 | 31.04 | 26.66 | 4.38 |
| *Superior temporal gyrus anterior part R* | -0.7768 | 0.1764 | 0.0001 | -0.0002 | 0.0002 | 0.1959 | 19.40 | 0.0003 | 41.62 | 27.51 | 14.11 |
| *Brainstem mid-brain B* | -1.4784 | 0.3885 | 0.0006 | 0.0003 | 0.0010 | 0.7324 | 14.48 | 0.0010 | 51.55 | 40.99 | 10.56 |
| *Brainstem pons B* | -1.4891 | 0.4329 | 0.0016 | 0.0006 | 0.0013 | 0.6181 | 11.83 | 0.0021 | 52.20 | 43.72 | 8.49 |
| *Brainstem medulla B* | -1.4615 | 0.4190 | 0.0014 | 0.0015 | 0.0010 | 0.1292 | 12.16 | 0.0019 | 53.05 | 44.30 | 8.76 |
| *Cerebellum gm R* | -1.1438 | 0.2312 | 0.0000 | 0.0000 | 0.0000 | 0.4533 | 24.48 | 0.0001 | 45.35 | 27.91 | 17.44 |
| *Cerebellum gm L* | -0.9904 | 0.2194 | 0.0001 | 0.0000 | 0.0000 | 0.1164 | 20.37 | 0.0002 | 44.21 | 29.44 | 14.77 |

Abbreviations: AIC - Akaike information criterion; ANOVA - analysis of variance; B – bilateral; FDR – false discovery rate; gm – grey matter; L – left; R – right.

**Supplementary Table 6.** **Group comparisons of regional residuals of** **[^11^C]UCB-J binding potential (BP_ND_) from data without partial-volume correction, obtained regressing out region-specific grey-matter volume.** P < 0.05 are highlighted in yellow, while p < 0.001 are highlighted in red.

|  | **BP_ND_ residuals** | | | | **Statistical parameters** | | | |  |
| --- | --- | --- | --- | --- | --- | --- | --- | --- | --- |
|  | **Mean CT** | **SD CT** | **Mean PT** | **SD PT** | **T value** | **Cohen's d** | **p** | **p FDR** | |
| *Hippocampus R* | 0.15 | 0.25 | -0.34 | 0.28 | -5.04 | -1.85 | 0.0001 | 0.0009 | |
| *Hippocampus L* | 0.12 | 0.24 | -0.27 | 0.27 | -4.07 | -1.51 | 0.0008 | 0.0039 | |
| *Amygdala R* | 0.09 | 0.33 | -0.21 | 0.21 | -3.31 | -1.10 | 0.0025 | 0.0071 | |
| *Amygdala L* | 0.09 | 0.32 | -0.21 | 0.19 | -3.53 | -1.15 | 0.0014 | 0.0049 | |
| *Anterior temporal lobe medial part R* | 0.09 | 0.30 | -0.21 | 0.23 | -3.28 | -1.12 | 0.0031 | 0.0082 | |
| *Anterior temporal lobe medial part L* | 0.10 | 0.28 | -0.22 | 0.22 | -3.69 | -1.27 | 0.0011 | 0.0045 | |
| *Anterior temporal lobe lateral part R* | 0.09 | 0.26 | -0.19 | 0.29 | -2.72 | -1.01 | 0.0143 | 0.0224 | |
| *Anterior temporal lobe lateral part L* | 0.08 | 0.26 | -0.17 | 0.24 | -2.79 | -1.00 | 0.0110 | 0.0196 | |
| *Parahippocampal and ambient gyri R* | 0.07 | 0.23 | -0.16 | 0.19 | -3.24 | -1.13 | 0.0036 | 0.0087 | |
| *Parahippocampal and ambient gyri L* | 0.05 | 0.24 | -0.12 | 0.17 | -2.47 | -0.84 | 0.0205 | 0.0298 | |
| *Superior temporal gyrus posterior part R* | 0.09 | 0.21 | -0.20 | 0.15 | -4.63 | -1.56 | 0.0001 | 0.0009 | |
| *Superior temporal gyrus posterior part L* | 0.09 | 0.22 | -0.22 | 0.16 | -4.81 | -1.64 | 0.0001 | 0.0009 | |
| *Middle and inferior temporal gyrus R* | 0.06 | 0.23 | -0.14 | 0.19 | -2.69 | -0.93 | 0.0128 | 0.0213 | |
| *Middle and inferior temporal gyrus L* | 0.06 | 0.28 | -0.14 | 0.21 | -2.32 | -0.80 | 0.0288 | 0.0396 | |
| *Fusiform gyrus R* | 0.14 | 0.29 | -0.33 | 0.26 | -4.89 | -1.72 | 0.0001 | 0.0009 | |
| *Fusiform gyrus L* | 0.12 | 0.29 | -0.28 | 0.29 | -3.89 | -1.41 | 0.0010 | 0.0041 | |
| *Insula L* | 0.08 | 0.23 | -0.17 | 0.25 | -2.82 | -1.03 | 0.0114 | 0.0199 | |
| *Insula R* | 0.09 | 0.24 | -0.20 | 0.24 | -3.28 | -1.19 | 0.0039 | 0.0088 | |
| *Lateral remainder of occipital lobe L* | 0.05 | 0.20 | -0.11 | 0.20 | -2.21 | -0.80 | 0.0397 | 0.0485 | |
| *Lateral remainder of occipital lobe R* | 0.04 | 0.18 | -0.09 | 0.19 | -1.97 | -0.72 | 0.0634 | 0.0698 | |
| *Cingulate gyrus anterior part L* | 0.08 | 0.30 | -0.19 | 0.26 | -2.84 | -1.00 | 0.0093 | 0.0171 | |
| *Cingulate gyrus anterior part R* | 0.13 | 0.27 | -0.30 | 0.30 | -4.05 | -1.50 | 0.0008 | 0.0039 | |
| *Gyrus cinguli posterior part L* | 0.15 | 0.23 | -0.34 | 0.27 | -5.17 | -1.93 | 0.0001 | 0.0009 | |
| *Gyrus cinguli posterior part R* | 0.13 | 0.23 | -0.29 | 0.28 | -4.32 | -1.62 | 0.0005 | 0.0033 | |
| *Middle frontal gyrus L* | 0.06 | 0.20 | -0.13 | 0.28 | -2.04 | -0.78 | 0.0597 | 0.0676 | |
| *Middle frontal gyrus R* | 0.06 | 0.20 | -0.13 | 0.26 | -2.15 | -0.82 | 0.0482 | 0.0571 | |
| *Posterior temporal lobe L* | 0.06 | 0.24 | -0.14 | 0.14 | -3.10 | -1.00 | 0.0040 | 0.0088 | |
| *Posterior temporal lobe R* | 0.04 | 0.20 | -0.08 | 0.15 | -2.00 | -0.69 | 0.0569 | 0.0654 | |
| *Inferiolateral remainder of parietal lobe L* | 0.07 | 0.21 | -0.15 | 0.24 | -2.64 | -0.98 | 0.0171 | 0.0259 | |
| *Inferiolateral remainder of parietal lobe R* | 0.08 | 0.18 | -0.18 | 0.23 | -3.32 | -1.25 | 0.0045 | 0.0090 | |
| *Caudate nucleus L* | 0.03 | 0.31 | -0.08 | 0.24 | -1.14 | -0.39 | 0.2661 | 0.2696 | |
| *Caudate nucleus R* | 0.02 | 0.30 | -0.06 | 0.15 | -1.07 | -0.34 | 0.2941 | 0.2941 | |
| *Caudate nucleus L* | 0.13 | 0.30 | -0.30 | 0.32 | -3.76 | -1.38 | 0.0014 | 0.0049 | |
| *Caudate nucleus R* | 0.13 | 0.34 | -0.30 | 0.37 | -3.26 | -1.20 | 0.0044 | 0.0090 | |
| *Putamen L* | 0.08 | 0.32 | -0.17 | 0.30 | -2.27 | -0.81 | 0.0336 | 0.0424 | |
| *Putamen R* | 0.07 | 0.31 | -0.17 | 0.28 | -2.28 | -0.81 | 0.0329 | 0.0423 | |
| *Thalamus L* | 0.06 | 0.22 | -0.15 | 0.27 | -2.25 | -0.85 | 0.0391 | 0.0485 | |
| *Thalamus R* | 0.07 | 0.22 | -0.16 | 0.24 | -2.71 | -1.00 | 0.0146 | 0.0224 | |
| *Pallidum L* | 0.08 | 0.20 | -0.19 | 0.19 | -4.02 | -1.44 | 0.0007 | 0.0038 | |
| *Pallidum R* | 0.08 | 0.20 | -0.18 | 0.19 | -3.87 | -1.39 | 0.0009 | 0.0041 | |
| *Precentral gyrus L* | 0.02 | 0.12 | -0.05 | 0.13 | -1.68 | -0.61 | 0.1109 | 0.1139 | |
| *Precentral gyrus R* | 0.03 | 0.12 | -0.07 | 0.14 | -1.99 | -0.74 | 0.0633 | 0.0698 | |
| *Straight gyrus L* | 0.11 | 0.37 | -0.24 | 0.24 | -3.43 | -1.13 | 0.0018 | 0.0058 | |
| *Straight gyrus R* | 0.13 | 0.35 | -0.30 | 0.23 | -4.40 | -1.46 | 0.0001 | 0.0011 | |
| *Anterior orbital gyrus L* | 0.06 | 0.19 | -0.14 | 0.34 | -1.83 | -0.73 | 0.0898 | 0.0935 | |
| *Anterior orbital gyrus R* | 0.05 | 0.19 | -0.12 | 0.26 | -1.94 | -0.74 | 0.0716 | 0.0756 | |
| *Inferior frontal gyrus L* | 0.09 | 0.28 | -0.20 | 0.22 | -3.41 | -1.18 | 0.0023 | 0.0070 | |
| *Inferior frontal gyrus R* | 0.08 | 0.22 | -0.19 | 0.24 | -3.17 | -1.16 | 0.0054 | 0.0104 | |
| *Superior frontal gyrus L* | 0.05 | 0.22 | -0.11 | 0.21 | -2.07 | -0.74 | 0.0514 | 0.0600 | |
| *Superior frontal gyrus R* | 0.04 | 0.19 | -0.10 | 0.22 | -1.93 | -0.71 | 0.0707 | 0.0756 | |
| *Postcentral gyrus L* | 0.04 | 0.14 | -0.08 | 0.12 | -2.65 | -0.93 | 0.0143 | 0.0224 | |
| *Postcentral gyrus R* | 0.04 | 0.14 | -0.08 | 0.12 | -2.70 | -0.95 | 0.0130 | 0.0213 | |
| *Superior parietal gyrus L* | 0.07 | 0.19 | -0.16 | 0.19 | -3.23 | -1.17 | 0.0044 | 0.0090 | |
| *Superior parietal gyrus R* | 0.07 | 0.17 | -0.16 | 0.20 | -3.35 | -1.25 | 0.0039 | 0.0088 | |
| *Lingual gyrus L* | 0.07 | 0.28 | -0.15 | 0.24 | -2.45 | -0.86 | 0.0228 | 0.0325 | |
| *Lingual gyrus R* | 0.06 | 0.25 | -0.14 | 0.25 | -2.15 | -0.78 | 0.0445 | 0.0536 | |
| *Cuneus L* | 0.10 | 0.29 | -0.22 | 0.27 | -3.17 | -1.13 | 0.0048 | 0.0095 | |
| *Cuneus R* | 0.11 | 0.29 | -0.25 | 0.28 | -3.46 | -1.24 | 0.0025 | 0.0071 | |
| *Medial orbital gyrus L* | 0.06 | 0.27 | -0.13 | 0.18 | -2.35 | -0.78 | 0.0263 | 0.0368 | |
| *Medial orbital gyrus R* | 0.06 | 0.28 | -0.14 | 0.23 | -2.32 | -0.81 | 0.0298 | 0.0402 | |
| *Lateral orbital gyrus L* | 0.06 | 0.23 | -0.14 | 0.25 | -2.33 | -0.86 | 0.0314 | 0.0416 | |
| *Lateral orbital gyrus R* | 0.10 | 0.24 | -0.22 | 0.27 | -3.37 | -1.26 | 0.0036 | 0.0087 | |
| *Posterior orbital gyrus L* | 0.05 | 0.26 | -0.12 | 0.19 | -2.26 | -0.77 | 0.0322 | 0.0421 | |
| *Posterior orbital gyrus R* | 0.07 | 0.25 | -0.15 | 0.24 | -2.54 | -0.92 | 0.0195 | 0.0288 | |
| *Cingulate rostral L* | 0.09 | 0.25 | -0.21 | 0.20 | -3.91 | -1.36 | 0.0007 | 0.0038 | |
| *Cingulate rostral R* | 0.12 | 0.28 | -0.27 | 0.24 | -4.30 | -1.51 | 0.0003 | 0.0020 | |
| *Cingulate rostral L* | 0.14 | 0.32 | -0.33 | 0.40 | -3.43 | -1.29 | 0.0035 | 0.0087 | |
| *Cingulate rostral R* | 0.17 | 0.38 | -0.38 | 0.43 | -3.68 | -1.36 | 0.0018 | 0.0058 | |
| *Cingulate rostral L* | 0.17 | 0.39 | -0.39 | 0.29 | -4.85 | -1.66 | 0.0001 | 0.0009 | |
| *Cingulate rostral R* | 0.23 | 0.31 | -0.52 | 0.35 | -6.09 | -2.26 | 0.0000 | 0.0009 | |
| *Superior temporal gyrus anterior part L* | 0.05 | 0.23 | -0.11 | 0.22 | -1.94 | -0.69 | 0.0669 | 0.0726 | |
| *Superior temporal gyrus anterior part R* | 0.11 | 0.28 | -0.24 | 0.25 | -3.71 | -1.31 | 0.0012 | 0.0048 | |
| *Brainstem mid-brain B* | 0.06 | 0.11 | -0.14 | 0.21 | -2.95 | -1.18 | 0.0119 | 0.0203 | |
| *Brainstem pons B* | 0.05 | 0.14 | -0.12 | 0.14 | -3.41 | -1.24 | 0.0030 | 0.0082 | |
| *Brainstem medulla B* | 0.06 | 0.13 | -0.13 | 0.17 | -3.14 | -1.20 | 0.0067 | 0.0126 | |
| *Cerebellum gm R* | 0.09 | 0.21 | -0.21 | 0.15 | -4.90 | -1.64 | 0.0000 | 0.0009 | |
| *Cerebellum gm L* | 0.09 | 0.23 | -0.21 | 0.18 | -4.23 | -1.45 | 0.0003 | 0.0020 | |

Abbreviations: B – bilateral; CT – controls; FDR – false discovery rate; gm – grey matter; L – left; PT – patients; R – right.
